# Hydration of Simple Model Peptides in Aqueous Osmolyte Solutions

**DOI:** 10.3390/ijms22179350

**Published:** 2021-08-28

**Authors:** Aneta Panuszko, Maciej Pieloszczyk, Anna Kuffel, Karol Jacek, Karol A. Biernacki, Sebastian Demkowicz, Janusz Stangret, Piotr Bruździak

**Affiliations:** 1 Department of Physical Chemistry, Gdańsk University of Technology, Narutowicza 11/12, 80-233 Gdańsk, Poland; vaskelion@gmail.com (M.P.); anna.kuffel@pg.edu.pl (A.K.); karol.jacek.pl@gmail.com (K.J.); janusz.stangret@pg.edu.pl (J.S.); piotr.bruzdziak@pg.edu.pl (P.B.); 2 Department of Organic Chemistry, Gdańsk University of Technology, Narutowicza 11/12, 80-233 Gdańsk, Poland; karbier1@gmail.com (K.A.B.); sebastian.demkowicz@pg.edu.pl (S.D.)

**Keywords:** osmolytes, small molecules, molecular chaperones, protein folding, protein misfolding, hydration water, FTIR spectroscopy, MD simulations

## Abstract

The biology and chemistry of proteins and peptides are inextricably linked with water as the solvent. The reason for the high stability of some proteins or uncontrolled aggregation of others may be hidden in the properties of their hydration water. In this study, we investigated the effect of stabilizing osmolyte–TMAO (trimethylamine *N*-oxide) and destabilizing osmolyte–urea on hydration shells of two short peptides, NAGMA (*N*-acetyl-glycine-methylamide) and diglycine, by means of FTIR spectroscopy and molecular dynamics simulations. We isolated the spectroscopic share of water molecules that are simultaneously under the influence of peptide and osmolyte and determined the structural and energetic properties of these water molecules. Our experimental and computational results revealed that the changes in the structure of water around peptides, caused by the presence of stabilizing or destabilizing osmolyte, are significantly different for both NAGMA and diglycine. The main factor determining the influence of osmolytes on peptides is the structural-energetic similarity of their hydration spheres. We showed that the chosen peptides can serve as models for various fragments of the protein surface: NAGMA for the protein backbone and diglycine for the protein surface with polar side chains.

## 1. Introduction

Many processes occurring in living organisms, e.g., protein folding, are vary labile in nature. Denaturation may lead to lower or even inhibited activity and can be linked with many physiological disorders. There are a lot of diseases that are caused by altered protein folding, for example Alzheimer’s disease, Parkinson’s disease, type 2 diabetes, and some prion diseases, which are collectively called amyloidosis. All of them are connected with the misfolding of peptides leading to their aggregation and formation of fibrils, which exhibit cytotoxicity [1,2,3,4].

Understanding the role of water in such processes is essential for better understanding of what happens inside living cells [5]. Especially its impact on the behavior of biomolecules requires detailed exploration. Apart from being a solvent, water molecules affect many processes, such as conformational stability, dynamics and enzymatic activity of proteins [6,7,8,9]. Its unique properties and influence on its surroundings results significantly from the ability to create hydrogen bonds. Being the natural environment for proteins or peptides, water is usually divided into three fractions: internal, hydration and bulk. The first one contains molecules that are confined inside proteins, and the second one contains hydration shells that can exchange water with the third one, which is the bulk and consists of free molecules. There are many studies focused on the impact exerted by proteins on water [10,11,12,13,14,15]. They reveal a slowdown in dynamics of hydration shells, compared to the bulk, especially rotational and translational motions in the first layer, accompanied by a longer hydrogen bond lifetime. Two of the cited works have shown that the slowdown is observed mainly near charged groups [14,15]. Other studies on short peptides have indicated that the dynamics of water are slower near hydrophilic moieties compared to hydrophobic ones [16,17,18,19,20]. To better investigate the properties of affected water, one needs to use models smaller than whole proteins because of a variety of interactions between many residues and chemical groups with the surrounding water. Using small model peptides, such as NAGMA (*N*-acetyl-glycine-methylamide or 2-acetamido-*N*-methylacetamide) or diglycine, helps to limit the amount of possible interactions and allows to avoid conformational changes of secondary or tertiary structures.

Small organic molecules called osmolytes are naturally used as one of the factors counteracting cellular stress, which can lead to protein denaturation or misfolding. Much research has been undertaken to study the influence of osmolytes on protein stability [21,22,23,24,25]. Urea is the one that is usually mentioned as a typical destabilizer, and TMAO (trimethylamine *N*-oxide) is known as one of the strongest protectants [26,27,28,29,30,31]. Despite numerous studies, there is still an active debate about the exact mechanism of the impact of osmolytes on protein folding. Various theories have emerged over many years. One of the most popular is based on direct osmolyte–protein interactions [21,22,23,32,33,34]. In accordance with this theory, stabilizers are preferentially excluded from the protein surface due to unfavorable interactions. This leads to a favorable hydration, thus increasing the stability of the native state. Denaturants in turn are attracted to the protein surface and backbone, thereby destabilizing the protein. Another often considered theory indicates an indirect mechanism based on changes in solvent properties caused by the presence of osmolytes [35,36,37,38,39,40,41,42]. Most of the stabilizing osmolytes enhance the water structure and can be classified as water structure-maker solutes. However, there are some exceptions in this group, such as taurine [43]. Similarly, in the group of destabilizers, there are compounds, mainly urea, whose influence on the water structure is still unclear [37,41,44,45,46]. Therefore, the structure making/breaking properties of osmolytes cannot always be attributed to their stabilizing/destabilizing effects [41,47].

As was pointed out before, short peptides are used to study hydration and its changes arising from the addition of osmolytes. Investigations with the use of NAAMA peptide (*N*-acetyl-alanine-methylamide or 2-(acetylamino)-*N*-methylpropanamide) indicated urea’s affinity to peptide bonds via hydrogen bonding [48]. Other studies have shown that urea substitutes water in the vicinity of the tryptophan moiety of the NATA peptide (*N*-acetyl–tryptophan-amide or 2-acetamido-3-(1*H*-indol-3-yl)propanamide) [49]. Stabilizers are usually excluded from the peptide surface causing stronger hydration [29,50,51,52].

In this paper, we investigate the impact exerted by stabilizing osmolyte–TMAO and denaturant–urea on hydration shells of two model peptides by means of the FTIR spectroscopy and MD simulations. NAGMA and diglycine peptides were chosen as the models for protein. Solutions were prepared according to the HDO (semi-heavy water) isotopic dilution method, ensuring quantitative interpretation. Analysis was performed by the difference spectra method. This allowed us to isolate the contribution of water molecules that are simultaneously under the influence of peptides and osmolytes. Further analysis led to the determination of structural and energetic properties of these water molecules.

## 2. Material and Methods

### 2.1. Chemicals and Solutions

FTIR experiment: diglycine (≥99%, Aldrich, Darmstadt, Germany), *N*-acetyl-glycine-methylamide (NAGMA, 2-acetamido-*N*-methylacetamide), prepared according to the method presented below), urea (99.5%, Aldrich, Darmstadt, Germany), trimethylamine-*N*-oxide dihydrate (≥99.0%, Sigma-Aldrich, Darmstadt, Germany) and deuterium oxide (isotopic purity 99.96%, Aldrich, Darmstadt, Germany) were used as supplied. Deionized water κ<0.01S·cm${}^{-1}$ was used for preparation of all solutions.

A series of aqueous solutions of peptide (NAGMA or diglycine)–osmolyte (TMAO or urea) systems containing different mole fractions of osmolyte (x_osm._ = 0.0, 0.2, 0.4, 0.5, 0.6, 0.8, 1.0) were prepared by weight. In this paper, the molar contribution of water will be ignored in mole fractions. These fractions indicate only molar ratios between peptide models and osmolytes. Other details of solution preparation for the HDO difference spectra method are described in our previous paper [53].

NAGMA synthesis: *N*-acetylglycine (99%, Sigma-Aldrich), methylamine hydrochloride (≥98%, Sigma-Aldrich), 1,1${}^{\prime }$-carbonyldiimidazole (CDI, reagent grade, ≥90%, Sigma-Aldrich), triethylamine (≥99.5%, Sigma-Aldrich) were used as supplied. Acetonitrile (MeCN), *N*,*N*-dimethylformamide (DMF), ethyl acetate were dried using standard procedures.

### 2.2. NAGMA Synthesis

NAGMA was prepared according to the procedure presented below:

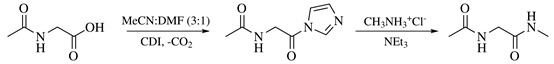



In a thoroughly dried round bottom flask, a mixture of CDI (1 eq, 0.107 mol, 17.35 g) in 200 mL of MeCN:DMF (3:1) was prepared and stirred under an argon atmosphere until CDI dissolved. Subsequently, *N*-acetylglycine (1 eq, 0.107 mol, 12.5 g) was introduced. After a few minutes, bubbles appeared, and the reaction mixture started to clear up. Next, the reaction was left under an argon atmosphere at room temperature for 3 h after the separation of bubbles had stopped. Afterward, methylamine hydrochloride (1 eq, 0.107 mol, 7.25 g) and triethylamine (2.1 eq, 0.225 mol, 22.72 g) were added, and the reaction mixture was sealed and left overnight at room temperature. Next, the solvent was evaporated to obtain yellowish oil, which was treated with 150 mL of AcOEt. The solution was refluxed for a few minutes. The boiling solvent was decanted and slowly cooled down. The precipitate was filtered and recrystallized from AcOEt to give pure product as a white solid (5.71 g) in 41% yield.

^1^H NMR (400 MHz, DMSO-d_6_, Figure S16) $\delta_{H}$ 8.08 (brs, 1H, NH), 7.75 (brs, 1H, NH), 3.61 (d, *J* = 5.8 Hz, 2H, CH_2_), 2.58 (d, *J* = 4.7 Hz, 3H, CH_3_), 1.85 (s, 3H, CH_3_); ^13^C NMR (101 MHz, DMSO-d_6_, Figure S17) $\delta_{C}$ 170.08, 169.85, 42.56, 25.90, 23.00; HRMS (Figure S18) *m*/*z*: [M + H]^+^ calculated for: C_5_H_10_N_2_O_2_ 130.0742; found 131.1145. Spectra were recorded on a Bruker AVANCE III HD 400 MHz spectrometer (Billerica, MA, USA). Chemical shifts δ are reported in parts per million relative to the residual solvent peak (DMSO-d_6_ = 2.51 ppm for ^1^H NMR and 39.5 ppm for ^13^C NMR). Coupling constants are reported in Hertz. The MS measurement was performed with TripleTOF 5600^+^ (SCIEX, Framingham, MA, USA).

### 2.3. FTIR Measurements

FTIR spectra of prepared solutions were recorded on Thermo Electron Co. Nicolet 8700 FTIR spectrometer (500–5000 cm^–1^, resolution of 4 cm^–1^, 128 scans for each spectrum). A liquid cell (model A145, Bruker Optics) with CaF${}_{2}$ windows separated with PTFE spacers was used. The path length was determined interferometrically (28.4 μm). The temperature of measurements was kept at 25 ± 0.1 and monitored using an electronic thermometer with thermocouples inserted into the cell. The spectrometer’s interior was purged with dry nitrogen to eliminate the influence of air components on a recorded spectra shape, namely, water vapor and carbon dioxide.

### 2.4. Spectral Data Analysis

The spectra were recorded and analyzed using the commercial PC software: OMNIC (Thermo Electron Corporation, Waltham, MA, USA), GRAMS/AI version 9.3 (Thermo Fisher Scientific Inc., Waltham, MA, USA) and RazorTools/8 (Spectrum Square Associates, Inc., Ithaca, NY, USA) run under GRAMS/AI.

The difference spectra method was used to extract the solute-affected HDO spectrum, extrapolated to the very diluted solution limit on the basis of spectra series, which were measured for different molalities of aqueous solutions. The method was described in detail in References [54,55,56]. This method assumes that water in a solution can be divided into two types: the “bulk” water (i.e., water exhibiting the properties of pure water) and “affected” water (i.e., water changed by interactions with the solute). The detailed procedure of spectral data analysis in systems containing two solutes has been previously described in Reference [53].

The solute-affected water spectra give valuable information about the energetic state of the hydrogen bonds in a selected population of water and intermolecular distances between water molecules in the vicinity of a solute. Details concerning the interpretation of the solute-affected water spectrum are included in the Supplementary Materials of Reference [41].

### 2.5. Molecular Dynamics Simulations

#### 2.5.1. Investigated Systems

Details of the preparation of the systems are given in Supplementary Materials [57,58,59,60,61,62]. We studied the systems consisting of:

- one diglycine molecule in water and one NAGMA molecule in water—these systems were used to find the most frequently occurring conformations of the solutes (through temperature-replica exchange molecular dynamics simulations) and to obtain trajectories (through molecular dynamics simulations) to study properties of solutes’ solvation shells (these included hydrogen bond analysis—described in more detail in the Supplementary Materials—and weak noncovalent interaction analysis—described below in Section 2.6). The solutes were placed in rectangular cuboid simulation boxes, with minimal distance from the box walls equal to about 20 Å. It ensured that the system remained relatively small but simultaneously big enough for the properties of solvation water to be able to converge to properties of bulk water with increasing distance from the peptide. The number of water molecules in these systems was equal to 4038.
- one diglycine molecule or one NAGMA molecule in water with three molecules of the osmolytes (TMAO or urea)—these systems were used to study interactions of the osmolytes with diglycine and NAGMA. They contained 4035 molecules of water. This corresponds to molal concentration equal to about 0.04 mol·kg${}^{-1}$.
- three TMAO or three urea molecules in water—these systems were used for the purposes of the initial assessment of the validity and applicability of the chosen sets of force field parameters. They contained 4040 molecules of water.

#### 2.5.2. Force Field Parameters

The force field used for proteins was ff03w [63], and the TIP4P/2005 [64] water model was employed. The ff03w force field is a popular Amber ff03 force field [65] with Best and Mittal modification [63]. It was optimized for the TIP4P/2005 water model. Since its introduction, this combination of parameters has been successfully used in studies by other researchers [66]. The TIP4P/2005 water model proved to be quite successful in predicting various properties of water [67,68], including some characteristics of the phase diagram [69].

The choice of force field parameters for the osmolytes is not straightforward because the available models receive mixed reviews. For urea, the parametrization available in Amber [70] was used for consistency. There is no consensus on the best representation of TMAO to be used in molecular dynamics simulations, and none of the models existing at the moment are ideal. We decided to use force fields parameters from Reference [71]. The applied set of parameters was tested by Markthaler et al. [72] in combination with the TIP4P/2005 water model, displaying adequate results.

#### 2.5.3. Protocols of the Simulations

Simulations were performed using the Amber 16 molecular dynamics package [70]. Prior to production runs, all systems were minimized and equilibrated; these procedures are described in more detail in the Supplementary Materials.

Temperature-replica exchange molecular dynamics simulations (REMD) were performed at 54 temperatures, ranging from 282.15 to 491.58 K. To generate this set of temperatures, an algorithm proposed by Patriksson and van der Spoel [59] was used. Amber 16 supports constant volume replica exchange simulations for explicit solvent. More details are given in the Supplementary Materials.

Molecular dynamics simulations were performed under NPT conditions (at 298 K, 1 bar). More details are given in the Supplementary Materials.

### 2.6. Weak Noncovalent Interaction Analysis

The most likely structures of these peptides in aqueous solutions are obtained as products of MD optimization and REMD simulations of the dynamics of NAGMA and diglycin structures in a water box. The structure of NAGMA surrounded by more than few water molecules has been proved previously to be stable [73]. This was facilitated by the relatively uncomplicated shape of these molecules. Next, the most likely structures were then placed inside the simulation box filled with TIP4P/2005 water. The positions of heavy atoms were restrained and a high force constant was applied (100 kcal·mol${}^{-1}$Å${}^{-2}$), (hence the need to initially obtain the most optimal structure), while the solvent molecules were allowed to move freely. The dynamics of such systems were simulated for 200 ps in steps of 0.002 ps, and from the obtained trajectories, only 1 frame per 100 was isolated to avoid situations where the positions of water molecules in two consecutive frames are very similar to each other. The obtained trajectories containing the largest possible representation of possible positions of solvent molecules around the peptides were transformed into averaged NCI functions (aNCI) using the Multiwfn 3.6 software [74]. These functions are created in a similar way to ordinary NCI functions [75,76] (RDG in older papers), but the electron densities around the atoms of the selected atomic subsystem (in our case of the central molecule and the nearest hydration layer) are obtained automatically by means of the so-called protomolecular approximation. The aNCI function with the help of the VMD 1.9.3 software enables the visualization of weak intermolecular noncovalent interactions. In our case, green denotes van der Waals interactions, and blue denotes hydrogen bonds. Importantly, the color intensity corresponds to the strength of the interaction, e.g., dark blue means strong hydrogen bonds, light blue or almost blue-green means very weak hydrogen bonds.

## 3. Results and Discussion

### 3.1. FTIR Investigation of Water Structure

#### 3.1.1. Characteristics of the Hydration Shell of Diglycine and NAGMA

**Figure 1:**
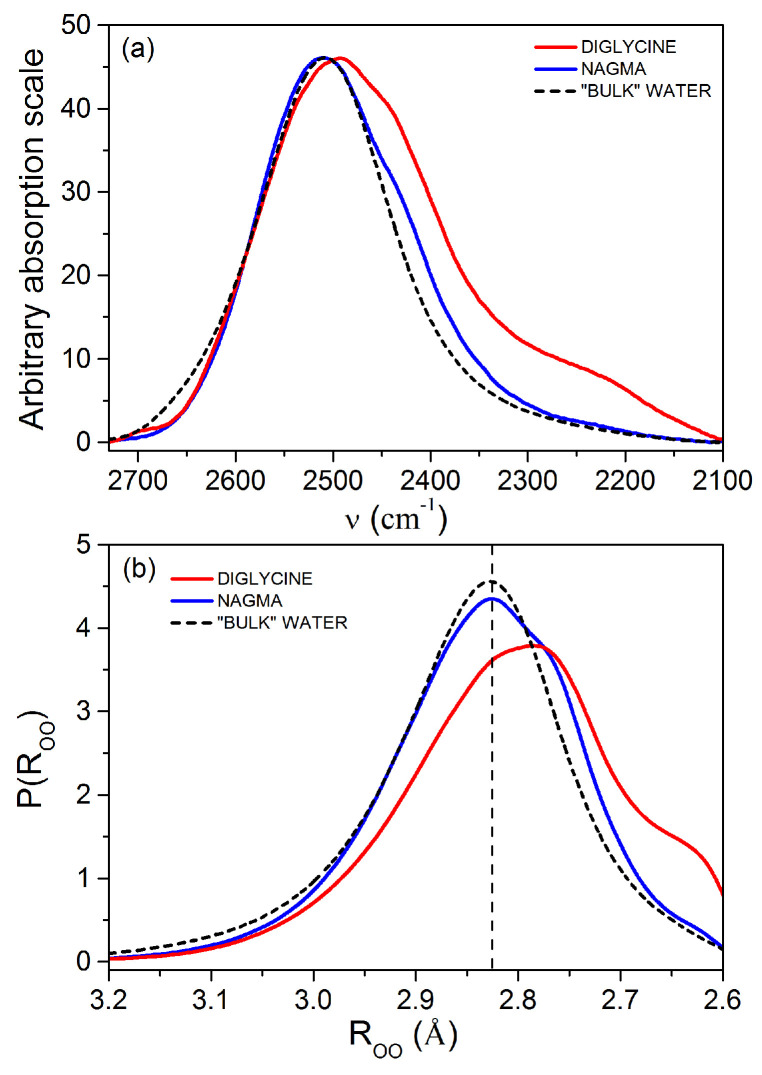
(**a**) Peptide-affected HDO spectra in the OD stretching region at the very diluted solution limit, along with the bulk HDO spectrum. The spectra have been scaled to the same maximum absorption value for better comparison. (**b**) Interatomic oxygen–oxygen distance distributions function obtained on the basis of the spectra shown in Figure 1a. The vertical dashed line corresponds to the value of the most likely oxygen–oxygen distance in bulk water (2.823 Å, see Table 1).

Figure 1a presents the peptide-affected HDO spectra and the “bulk” water spectrum. For better comparison, the height of all spectra has been scaled to the same height. These spectra were transformed into the oxygen–oxygen distance distribution functions, $P(R_{OO})$, as described in Reference [56]. The obtained distance probability distributions are shown in Figure 1. Spectral parameters of peptide-affected HDO bands and the bulk HDO band, together with intermolecular oxygen–oxygen distances, $R_{OO}$, are presented in Table 1.

Based on the confrontation of the spectral contours (Figure 1), only slight differences can be seen between NAGMA-affected water and pure water spectral contours, while highly polar diglycine affects the shape of the OD band significantly. Analysis of the values of the mean oxygen–oxygen distances $\left(R_{OO}^{g}\right)$ for peptide-affected water and for “bulk” water (Table 1) points out that water–water hydrogen bonds are shorter in the presence of both of these peptides. Moreover, water affected by NAGMA has the same value of the most likely oxygen–oxygen distance as in pure water. The shift in the values of the OD band gravity center, $\nu_{OD}^{g}$ (related to the mean energy of water hydrogen bonds), towards lower values with respect to the ones corresponding to pure water (Table 1), indicates that water molecules surrounding the peptides form on average stronger H-bonds than in pure water. In addition, hydrogen bonds of water molecules around diglycine are stronger and shorter than those in water affected by NAGMA.

The analysis of oxygen–oxygen distance distributions, $P(R_{OO})$, (Figure 1b) shows that the probability of strong hydrogen bond formation increases in the nearest surroundings of the peptides ($R_{OO}\approx 2.75$ Å) relative to bulk water. A higher number of strong hydrogen bonds in the hydration sphere of diglycine is observed. Simultaneously, the contributions of water molecules with very weak hydrogen bonds ($R_{OO}\geq 3.0$ Å) and those with the mean energy of hydrogen bonds (distances equal and only slightly longer than to the most probable distance in bulk water, value $R_{OO}=2.823$ Å, Table 1) in the presence of peptides decrease. The share of water molecules with mean energy of H-bonds in NAGMA-affected water is only slightly lower than in pure water.

The obtained structural and energetic characteristics of water molecules affected by peptides show that both peptides enhance the water structure in their nearest surrounding (relative to pure water). However, in the case of NAGMA, this effect is less pronounced.

#### 3.1.2. Analysis of the Water Structure around Peptides in the Presence of Osmolytes

The spectra of water changed by the simultaneous presence of two solutes in the mixture (peptide and osmolyte) will be called the experimental spectra of affected water. Such spectra contain parts corresponding to water populations affected by each solute separately (i.e., peptide-affected water and osmolyte-affected water) and those water molecules that are simultaneously under the influence of both peptides and osmolytes. Theoretical spectra of affected water will be used as references for the analysis of experimental spectra. They are constructed as follows: spectral contours of the water affected by pure components (i.e., peptide and osmolyte) are added together in an appropriate proportion taking into account the numbers of water molecules affected by them and the ratio of the number of moles of both solutes in the solution (see Table S5 in Supplementary Materials). A detailed description of theoretical spectra preparation can be found in Reference [53]. The theoretical spectra reflect a hypothetical situation in solution when both solutes influence water, but they or their hydration spheres do not interact with each other.

Experimental and theoretical spectra in the peptide–osmolyte systems are presented in Figures S1 and S2 in the Supplementary Materials, respectively. The corresponding numbers of affected water molecules and the differences between them (*N*_exp._-*N*_theor._) are presented in Table S5 in the Supplementary Materials. The distance probability distributions obtained from the transformation of these spectra are shown in Figures S3 and S4 in the Supplementary Materials. All parameters of experimental affected water, water affected by pure solutes, bulk water and theoretical affected water, together with the intermolecular oxygen–oxygen distances, $R_{OO}$, are summarized in Tables S1–S4 (Supplementary Materials).

**Figure 2:**
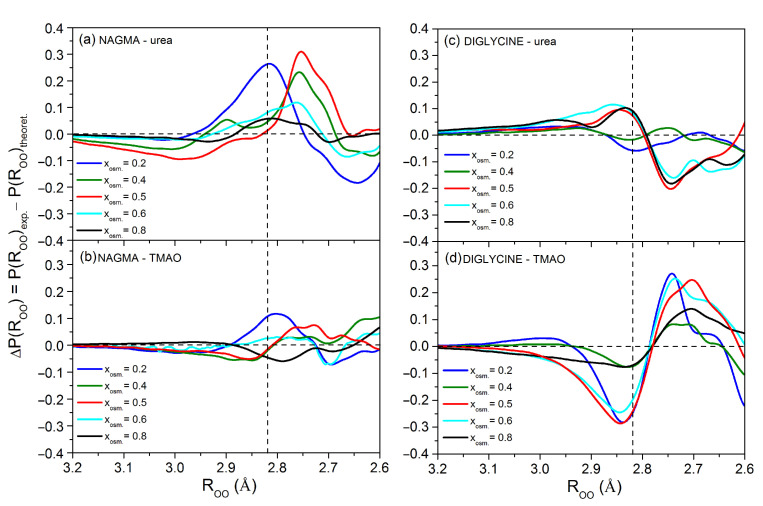
Differences between experimental (from Figure S3), $P{\left(R_{OO}\right)}_{\exp.}$ and theoretical (from Figure S4), $P{\left(R_{OO}\right)}_{\mathrm{theoret}.}$ distributions of interatomic oxygen–oxygen distances for (**a**,**b**) NAGMA–osmolyte and (**c**,**d**) diglycine–osmolyte systems. The vertical dashed line corresponds to the value of the most likely oxygen–oxygen distance in bulk water (2.823 Å, see Table 1).

The differences in the distribution of oxygen–oxygen distances between experimental and theoretical affected water, $\Delta P(R_{OO})$, (Figure 2) show changes in distances between water molecules when the hydration spheres of both solutes interact with each other in relation to the non-interacting hydration spheres of both solutes in a hypothetical solution. They provide general information about the mutual influence of both solutes: whether there is an influence and what its character is. Thus, any discrepancy between the experimental and theoretical distributions is evidence that the two solutes interact with each other. It should be noted that as a result of the interaction of the hydration spheres of both solutes, not only the properties of water molecules directly involved in this interaction change but also those around each solute.

The differences $\Delta P(R_{OO})$ obtained for the NAGMA–urea system (Figure 2a) show that even the smallest addition of urea to the NAGMA solution (x_urea_ = 0.2) increases the contribution of water molecules with the mean energy of hydrogen bonds (distances equal and slightly shorter and longer than to the most likely distance in bulk water, value $R_{OO}=2.823$ Å, Table 1) in the experimental water compared to the theoretical water. For x_urea_ = 0.4 and 0.5, the contact of the hydration spheres of both solutes favors shorter distances between water molecules. Interestingly, with an excess of urea in the solution (x_urea_ = 0.6 and 0.8), the differences in $\Delta P(R_{OO})$ are very small, indicating that the interactions of the hydration spheres of both solutes are poor.

Only slight differences between the experimental and theoretical water can be observed for the NAGMA–TMAO system (Figure 2b). This means that the contact of the hydration spheres of both molecules is rather rare. However, the smallest addition of TMAO to the solution (x_TMAO_ = 0.2) has a similar effect to that observed in the NAGMA–urea system, but in this case, it is less pronounced. At this stage of our research, it is difficult to explain such a behavior of NAGMA systems where the peptide is in excess. However, we can speculate that competition between NAGMA molecules for free osmolyte molecules occurs in such solutions. It can be imagined that NAGMA molecules try to steal osmolytes from hydration spheres of other peptides in solution and thus weaken the energies of the overall NAGMA–osmolyte interaction.

The case of the diglycine–urea system (Figure 2c) is different. Here, small urea additions (x_urea_ = 0.2 and 0.4) cause only slight changes of $\Delta P(R_{OO})$. It proves that interactions of the peptide and osmolyte hydration spheres are weak. In turn, for urea mole fractions from 0.5 to 0.8, the contact of the hydration spheres of both molecules causes a decrease in the population of strong hydrogen bonds and an increase in the population of both the mean and the weaker hydrogen bonds between water molecules. It is worth noting that for these molar fractions, positive values of Δ*N* (Table S5 in Supplementary Materials), calculated as the difference between the experimental and theoretical number of affected molecules, were obtained. This result indicates that additional water molecules are affected, the so-called excess water molecules. For the remaining systems, negative values of *N* are observed, which means that some affected water molecules are under the influence of both solutes simultaneously, i.e., they are shared water molecules in the hydration spheres of both solutes.

The interactions between the hydration spheres of diglycine and TMAO (Figure 2d) are more likely than in the NAGMA–TMAO system and cause an increase in the contribution of strong water hydrogen bonds and a decrease in the contribution of water molecules with mean and weak energy of hydrogen bonds in the entire range of TMAO mole fractions.

The above analysis of the differences in the distribution of distances between the experimental and theoretical affected water shows that urea interacts more frequently with NAGMA, which resembles the protein backbone, while TMAO has a greater effect on diglycine, which is more similar to the protein surface with polar side chains.

#### 3.1.3. Water Spectra Changed Simultaneously by Peptide and Osmolyte

The spectral shape of the whole water population affected by two solutes in a ternary solution is not the only information we are able to obtain out of the spectral data. With our recently developed method of analysis [53], we can extract spectra of these populations of water molecules, which are simultaneously under the influence of both peptides and osmolytes, i.e., shared or excess water molecules. Both shared or excess affected water is water affected simultaneously by both solutes. Shared water is formed by the overlapping of the hydration spheres of both solutes. Excess affected water results from: (1) affecting additional water molecules in the hydration shells of solutes (i.e., these water molecules were not considered affected before the contact) or (2) water molecules connecting the hydration spheres of both solutes, the so-called bridging water, which come from the bulk surrounding solvent.

##### Spectral Share Analysis

**Figure 3:**
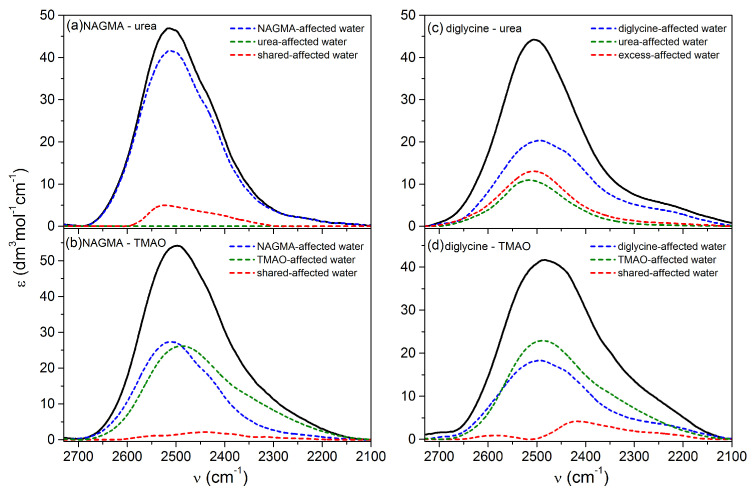
The spectra of water affected by peptide and osmolyte in a mixture (experimental affected spectra, from Figure S1) for (**a**,**b**) NAGMA–osmolyte and (**c**,**d**) diglycine–osmolyte systems for 1:1 mole fraction with separated contribution of shared or excess affected water, peptide-affected water and osmolyte-affected water.

Figure 3 shows the experimental affected spectra for the 1:1 molar ratio with the extracted contribution of shared or excess affected water and the spectral share of water affected solely by pure solutes. The spectra for the remaining mole fractions are shown in Supplementary Materials in Figures S6–S9.

The contribution of shared water in the NAGMA–urea system for a 1:1 molar ratio (Figure 3a) is ≈10% in the experimental spectrum and is a mixture of different populations of hydrogen-bonded water. Interestingly, there is no water affected by pure urea in the experimental spectrum, which suggests that all affected water molecules around the urea were included in the NAGMA hydration sphere. Therefore, the shared water contribution arises mainly from the urea hydration sphere. It can be said that urea with its hydration sphere blends into the hydration sphere of the peptide. This may indicate a direct interaction of urea with the NAGMA surface. The urea affinity to the peptide bond was also proved in [48] on a very similar peptide NAAMA and for the backbone of the NATA peptide [49]. A similar effect is observed for x_urea_ = 0.4 (Figure S5b in Supplementary Materials). The smallest contribution of shared water in the experimental spectrum (≈1.2%) occurs when the urea:peptide ratio is high (x_urea_ = 0.8). In this case, the contact of the hydration spheres of both solutes is insignificant, which is also suggested by the differences in the distribution of distances between the experimental and theoretical affected water (Figure 2a).

Very small contributions of shared affected water are observed for the NAGMA–TMAO system in the entire range of TMAO mole fractions (Figure S6 in Supplementary Materials). Its contribution in the experimental spectrum for TMAO mole fractions from 0.2 to 0.6 equals ca. 2.4%, while for x_TMAO_ = 0.8 it is only ca. 0.6%. This observation confirms that the interaction of the NAGMA and TMAO hydration spheres is poor in the entire composition range.

In the case of the diglycine–urea 1:1 system (Figure 3c), the contribution of excess water in the experimental spectrum is exceptionally high (≈27%). The situation is similar for x_urea_ = 0.6 and 0.8 (Figure S7d,e in Supplementary Materials). The properties of excess water molecules are similar to those for pure water. This can be seen in the inset in Figure S9b in the Supplementary Materials, where a comparison of the oxygen–oxygen distance distributions for excess and bulk water is shown. This is also reflected in the population of hydrogen bonds with distances equal to and longer than the most probable distance in bulk water due to the interaction of both solutes than in the absence of contact of the hydration spheres (i.e., in theoretical water) (Figure 2c). The small contribution of shared water with the smallest urea additions x_urea_ = 0.2 and 0.4 (Figure S7a,b in Supplementary Materials) confirms that the contact of diglycine and urea hydration spheres is insignificant.

Shared water in the diglycine–TMAO system for 1:1 molar ratio (Figure 3d) is characterized by two populations of hydrogen bonds: a very small share of weak hydrogen bonds (at ca. 2590 cm^–1^) and a significant share of very strong hydrogen bonds of water (at ca. 2416 cm^–1^). These two populations are present in the entire range of TMAO mole fractions (Figure S8 in the Supplementary Materials). The largest contribution of shared water in the experimental spectrum occurs at the lowest TMAO content in the solution (x_TMAO_ = 0.2), which indicates a strong influence of TMAO on the diglycine hydration shell.

##### Structural-Energetic Characteristics of Shared/Excess Affected Water

Contours of the spectra of shared and excess affected water for a 1:1 molar ratio are presented in Figure S9a (Supplementary Materials). The oxygen–oxygen distance distributions, obtained by a transformation of these contours, are shown in Figure S9b in the Supplementary Materials. The spectral parameters of shared and excess affected water for x_osm._ = 0.5, peptide-affected water, osmolyte-affected water and bulk water, together with the intermolecular oxygen–oxygen distances, $R_{OO}$, are presented in Table 1.

**Figure 4:**
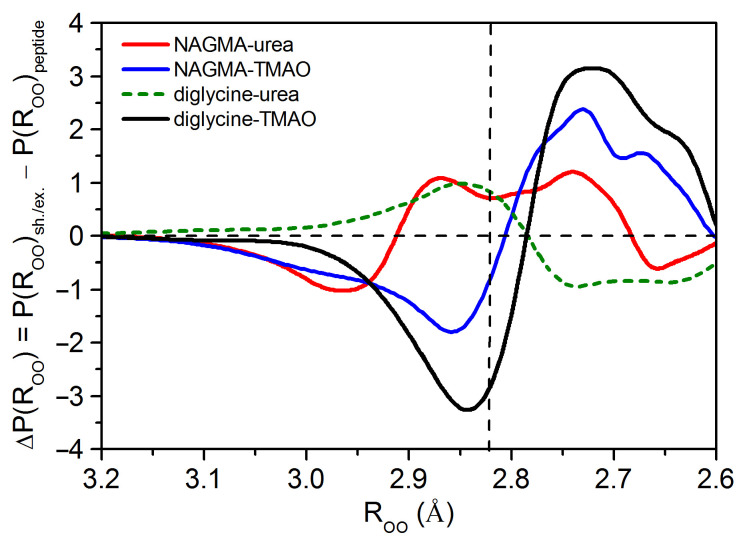
Differences between the interatomic oxygen–oxygen distance distribution function of water affected simultaneously by peptide and osmolyte for 1:1 mole ratio (solid line for shared affected water and dashed line for excess affected water, from Figure S9b), $P{\left(R_{OO}\right)}_{\mathrm{sh}./\mathrm{ex}.}$, and the peptide affected water (from Figure 1b), $P{\left(R_{OO}\right)}_{\mathrm{peptide}}$. The vertical dashed line corresponds to the value of the most likely oxygen–oxygen distance in bulk water (2.823 Å, see Table 1).

Detailed information is provided by the differences in the oxygen–oxygen distance distributions between shared/excess water and peptide-affected water (Figure 4). They illustrate how the distances between water molecules under the simultaneous influence of both solutes in solution have changed in relation to the peptide hydration sphere. It is worth noting that the comparison of the water around a central particle with the water around it in the presence of an additional co-solute appears quite often in the literature. However, it is often forgotten in such studies that the mere introduction of an additional substance with its hydration shell radically changes the average picture of hydration in such systems but does not necessarily mean a change in the hydration shell of the central molecule. For example, when a solution with a substance A, which strongly weakens water, is added to some other solute B, which strongly strengthen this water, an average image of water with properties similar to bulk water can be pictured.It can be mistakenly concluded that the additional substance B strengthens the hydration shell of the basic substance A and makes its properties more like in the bulk water. However, both substances can be completely independent in solution, as can their hydration shells. Interactions between the solutes and their hydration shells must be proved each time so that such conclusions about the mutual influence on hydration shells of two solutes can be drawn.

The presence of TMAO in peptide solutions, both in the NAGMA–TMAO and diglycine–TMAO systems, reduces the population of water molecules with the average energy of a hydrogen bond (distances equal to and slightly longer than the most likely distance in bulk water) and increases the population of water molecules with the strong energy of hydrogen bonds (short O⋯O distances) compared to the water around diglycine (Figure 4). This effect is more pronounced in the diglycine–TMAO system. The analysis of the differences in the oxygen–oxygen distance distributions for diglycine-affected water and TMAO-affected water with respect to the bulk water shows that they are very similar: both water around TMAO and water around diglycine are characterized by the population of very strong hydrogen bonds (Figure S10a). It has been shown that the hydration sphere of stabilizing osmolytes is strikingly similar to the hydration sphere of lysozyme [41]. TMAO generates very strong hydrogen bonds in its surrounding, much stronger than in the bulk water. In this sense, TMAO enhances the water structure, which is consistent with other studies [36,77]. Furthermore, the mean energy of hydrogen bonds in TMAO-affected water is comparable to that in the hydration sphere of diglycine (Table 1). The interaction of the reinforced hydration shells of both solutes leads to an additional strengthening of hydrogen bonds in the shared-affected water when compared to pure water (Figure S10b). As a result, water molecules in the shared water form stronger hydrogen bonds than in the hydration shell of diglycine (Figure 4).

In the case of the NAGMA–TMAO system, the interaction of the hydration spheres of both solutes is insignificant (confirmed by the differences in $\Delta P(R_{OO})$ in Figure 2b). The mutual contact of the strongly enhanced TMAO hydration sphere and, to a lesser extent, the enhanced NAGMA hydration sphere (Figure S10a) increases the contribution of water molecules with shorter distances in the shared-affected water relative to the bulk water (Figure S10b), and thus relative to the NAGMA hydration layer (Figure 4).

The fact that in systems with TMAO-affected water molecules are shared between hydration spheres of co-solutes (i.e., hydration spheres are overlapped) allows the assumption that TMAO accumulates at the surface of both peptides. This observation is consistent with that obtained from the work of Liao et al. [78]. TMAO stabilizes folded conformations because, thanks to its amphiphilic character, it is efficient in interacting with the protein surface where polar and nonpolar regions can be found in close proximity. They observed some accumulation of TMAO in the vicinity of a polypeptide, in contrast to other osmolytes: glycine and betaine. A similar effect was also observed in the proline–lysozyme system [79]. According to the theory of preferential exclusion, stabilizing osmolytes should be excluded from the surface of proteins [21,34,36].

Urea has a different effect on peptides than TMAO. Moreover, the changes in the hydration spheres of both peptides due to the presence of urea are different. The properties of urea-affected water are similar to those of bulk water. The mean energy of hydrogen bonds and the average distance between the water molecules in the urea-affected water is the same as in pure water (Table 1). However, slight differences in the populations of water molecules around urea compared to bulk water can be noticed: water affected by urea shows a slightly higher population of water molecules with distances equal to and longer than the most likely distance in pure water and a certain population of shorter ones (Figure S10a). Some of the previous studies also show that urea has a negligible effect on the structure of water [44,45], while in others, it is termed as a structure breaker [37,46]. Moreover, urea and its alkyl derivatives, despite their ability to denature proteins, enhance the water structure, although urea influences it rather weakly [41]. The introduction of urea to the diglycine solution disturbs additional water molecules in the system and makes them more similar to the bulk water (Figure S10b). The occurrence of excess water molecules and their resemblance to pure water may indicate the exclusion of urea from the hydration sphere of diglycine. In light of other studies, it is believed that destabilizing osmolytes, such as urea, interact directly with protein [33,80]. As a result, in excess-affected water, we observe an increase in the contribution of water molecules with the average energy of a hydrogen bond and a decrease in the contribution of strong hydrogen bonds in relation to the diglycine hydration sphere (Figure 4). Moreover, the excess affected water molecules form, on average, weaker hydrogen bonds than in the diglycine hydration sphere (Table 1).

Based on the differences in the distance distributions in relation to bulk water for urea-affected water and NAGMA-affected water (Figure S10a) and for shared-affected water in NAGMA–urea system (Figure S10b), it can be noticed that urea contributes to the NAGMA hydration shell a population of water molecules with distances equal to and longer than the most likely distance in bulk water and a smaller population of strong hydrogen bonds. It should be noted that in this system, all affected water molecules in the hydration sphere of urea are blended into the NAGMA hydration shell (see discussion in Section 3.1.3). In effect, the shared-affected water in the NAGMA–urea system is characterized by a population of hydrogen bonds with distances equal to and longer than the most likely distance in pure water and those shorter in relation to the NAGMA hydration sphere (Figure 4). However, the contribution of the shorter ones is slightly higher and, on average, hydrogen bonds in the shared-affected water in this system are slightly stronger than in the water affected by NAGMA (Table 1). Apart from this small enhancement, there is a growth in the amount of bulk-like water molecules in the shared-affected water. A higher contribution of this fraction on the protein surface makes them easier to aggregate [81].

### 3.2. Noncovalent Interaction Analysis in Peptide–Water Systems

In the case of both peptides, the difference in the shape of the van der Waals interaction surfaces around the hydrophobic groups is immediately visible. In NAGMA, methyl groups are surrounded by a dense shell of weak interactions, and it can be assumed that the surrounding water molecules form a typical hydrophobic cage. Interactions of this type create a closed cocoon pierced only in the places where typically hydrophilic groups occur. However, due to the fact that they are relatively isolated from each other, there is no cooperation between them, and they are weaker than analog places in diglycine (this is evidenced by a less intense color in the places where hydrogen interactions occur). In the case of diglycine, there are no such strong centers to promote hydrophobic hydration, hence the sphere of van der Waals interactions in this dipeptide is jagged and not as compact as in the case of NAGMA. Interactions with polar groups dominate, which clearly proves that the dominant type of hydration is hydrophilic hydration. Furthermore, one can immediately see large spaces of interactions of the carboxyl group with water molecules in the form of red-blue circles, the proximity of which also strengthens the interaction of the closest N–H group with water than in similar interaction sites in the NAGMA molecule (blue is darker in the case of diglycine than in NAGMA). Despite the large distance of the carbonyl bond from the dominant carboxyl group, its hydrogen bonds with water molecules are stronger than in NAGMA (intense colors of the interaction space) and are better oriented, i.e., in the case of NAGMA, the place of such interaction is wide and diffuse, while in diglycine it is well oriented at an angle of approx. 30° from the bond axis of C=O group. This better orientation in diglycine occurs even in the absence of the potential steric hindrance, which certainly occurs due to the presence of the CH_3_ group at a similar site in the NAGMA molecule.

### 3.3. Molecular Dynamics Simulations

The analysis of the results of the molecular dynamics simulations began with an initial assessment of the validity and applicability of the chosen force field parameters. To this end, the distributions of the lengths of hydrogen bonds in solvation shells of TMAO molecules, urea molecules, NAGMA and diglycine were computed and compared with the conclusions drawn experimentally. The details of these calculations together with the respective figures can be found in the Supplementary Materials. The results of the molecular dynamics simulations reproduce approximately the trends observed experimentally.

The differences in degree of hydrophobic character of solvation of diglycine and NAGMA discussed in Section 3.2 can be supported by the analysis of number of hydrogen bonds in solvation shells (Figure S15). In the solvation shell of NAGMA, a molecule of water creates more hydrogen bonds with other water molecules than it would if the structure of this water was strictly bulk-like. In a solvation shell of diglycine, on the other hand, water molecules closest to its surface create fewer hydrogen bonds with each other, which suggests that they are instead hydrogen-bonded to the peptide.

**Figure 5:**
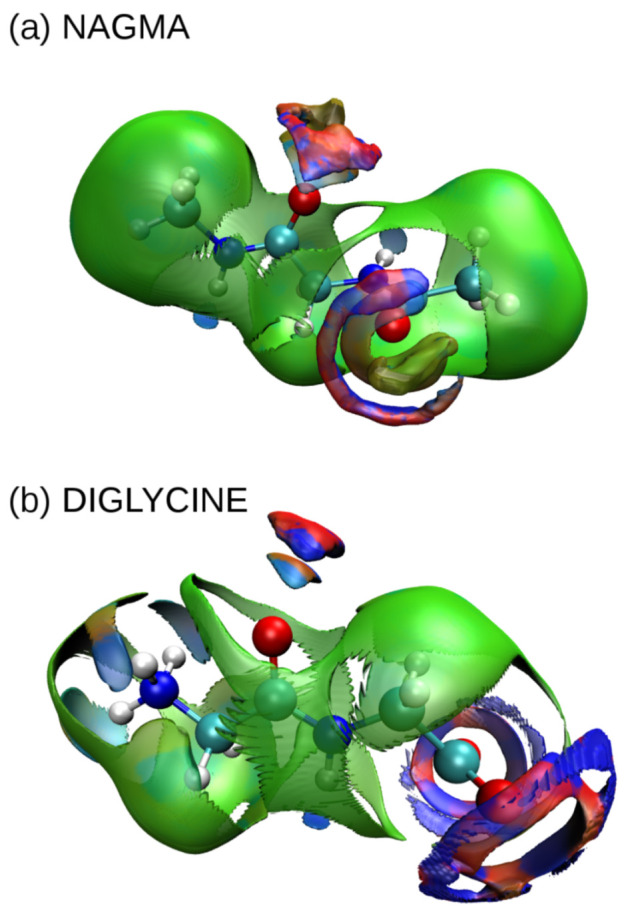
Visualization of aNCI functions for (**a**) NAGMA and (**b**) diglycine placed in a box of TIP4P/2005 water. Green patches denote van der Waals interactions, blue or blue/red hydrogen bonds.

**Figure 6:**
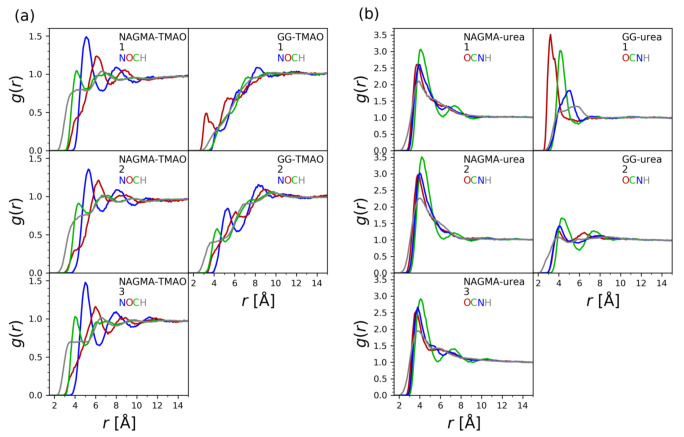
Radial distribution functions of atoms of the osmolyte molecules (urea and TMAO) calculated separately for geometric centers of three parts of NAGMA molecule (1. *N*-acetyl, 2. glycine and 3. methylamide groups) and two parts of diglycine molecule (1. N-terminal glycine, 2. C-terminal glycine). In (**a**), the distributions of TMAO atoms are presented, in (**b**), the distributions of urea atoms are presented.

As the results presented in Figure 5 suggested, water organizes itself approximately uniformly with respect to the whole NAGMA molecule. The results of molecular dynamics simulations indicate that the same may be true when it comes to the osmolyte molecules. NAGMA molecule can be divided into three parts: *N*-acetyl, glycine an methylamide groups. We determined the distances of all atoms constituting the osmolyte molecules relatively to the geometric center (GC) of each of these three groups and calculated radial distribution functions according to the well-known relation $g\left(r\right)=\frac{V}{N}\cdot \frac{dN}{dV}$, where *N* was the number of respective atoms of osmolytes and *V* was the volume (Figure 6).

In the systems consisting of NAGMA and urea, urea molecules are observed to be organized around each part of the NAGMA molecule in the same manner. The distributions of distances corresponding to each atom of the osmolyte almost overlap, suggesting that urea molecules do not preferentially direct any of their atoms towards the NAGMA molecule. Tall peaks in Figure 6 suggest that urea tends to accumulate around NAGMA.

**Figure 7:**
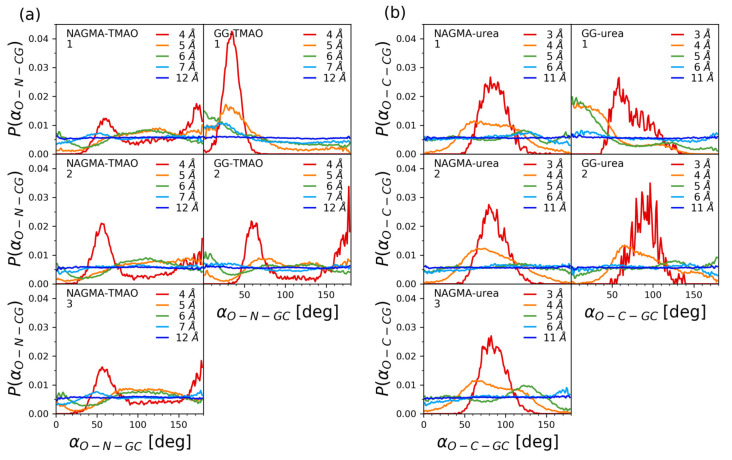
Distributions of angles O–C–GC and O–N–GC characterizing the orientation of the osmolyte molecules (urea and TMAO) relative to the NAGMA and diglycine molecules, calculated separately for geometric centers (GC) of three parts of the NAGMA molecule (1. *N*-acetyl, 2. glycine and 3. methylamide groups) and two parts of the diglycine molecule (1. N-terminal glycine, 2. C-terminal glycine). Angle O–C–GC is measured as an angle between the following vectors: one running through C and O atoms of urea a molecule, and the other pointing from the C atom of urea to the geometric center of a selected part of the NAGMA or diglycine molecule. Angle O–N–GC is measured as an angle between the following vectors: one running through N and O atoms of the TMAO molecule, and the other pointing from the N atom of TMAO to the geometric center of selected part of the NAGMA or diglycine molecule. In (**a**), the distributions of angles measured for TMAO are presented, in (**b**), the distributions of angles measured for urea are presented. The angles are measured for various distances between C or N atoms (of urea or TMAO) and geometric centers (GCs) of the respective groups of NAGMA or diglycine (given in the legends).

To additionally investigate the orientation of the osmolytes relative to the NAGMA molecule, the angles describing the orientations of the C=O bond of the urea molecule were analyzed. They were measured between two vectors. The first vector was defined by the C=O bond of the urea molecule, and the second vector was connecting the carbon atom of the urea molecule with the geometric centers (GCs) of the three groups into which the molecule of NAGMA was divided. The distribution of these angles is presented in Figure 7. All of these distributions were normalized to unit area.

The distribution of O–C–GC angles do not change significantly between the three groups of atoms constituting the NAGMA molecule, supporting the conclusions expressed above, concerning roughly uniform interactions of osmolytes with each part of the NAGMA molecule. The O–C–GC angles, together with the radial distribution functions, allow to hypothesize the preferred orientation of the urea molecules relative to the NAGMA molecule. At the shortest distances of the carbon atom of urea from the geometric centers of the three groups of NAGMA molecule, distinct peaks with a maximum at about $90^{\circ }$ are observed, suggesting a parallel orientation of the flat surface of the urea molecule (defined by the bonds created by the carbon atom) relative to the surface of the NAGMA molecule. Because of the geometry of the urea molecule, this orientation of the osmolyte enables the carbon atom of urea to approach the NAGMA molecule the closest; therefore, it is not surprising that these angles are favored at the shortest distances. However, some preference for angles equal to about $90^{\circ }$ paired with the avoidance of very small or very large angles (the avoidance of perpendicular orientation of a urea molecule relative to the surface of NAGMA) is still visible slightly farther from the NAGMA molecule, before the distribution becomes uniform. This kind of more or less parallel orientation of urea molecule (together with the above-mentioned tendency to accumulate in close proximity of the NAGMA molecule) might potentially facilitate the submersion of a solvation shell of urea in a solvation shell of NAGMA observed experimentally for lower mole fractions of urea.

The accumulation of TMAO in close proximity to NAGMA, as deduced from the values of radial distribution functions in Figure 6, is significantly smaller compared to urea. TMAO molecules also orient themselves approximately uniformly around the whole NAGMA molecule, except this time the distributions of distances corresponding to the atoms constituting TMAO molecule do not overlap as in the case of urea (Figure 6). The order of the observed maxima suggests that TMAO molecules that are close to the NAGMA molecule tend to orient themselves with their methyl groups facing the NAGMA molecule. This is consistent with partly hydrophobic character of the solvation layer of NAGMA and also not conflicted with, observed experimentally, the smaller influence of TMAO than urea on the hydration layer of NAGMA. Additionally, the angles O–N–GC describing the orientation of the N–O bond of TMAO relative to the NAGMA molecule were analyzed analogically to the O–C–GC angles discussed above for the NAGMA—urea system (Figure 7). They confirm the conclusion regarding similar orientations of the TMAO molecules near each part of the NAGMA molecule.

The solvation shell of diglycine was found to be inhomogeneous (Figure 5). Analogically, the results of molecular dynamics indicate that the osmolytes (both TMAO and urea) interact differently with the first (N-terminal) and the second (C-terminal) glycine residue.

While urea accumulates readily around the whole NAGMA molecule, in the case of diglycine, it displays comparable affinity only towards the first, positively charged, N-terminal part of the molecule (Figure 6). When close to the N-terminal glycine, the molecules of urea preferentially orient their oxygen atoms towards the dipeptide. An analogical situation occurs when TMAO is present in the system. TMAO molecules near the N-terminal glycine tend to direct their oxygen atoms towards the dipeptide, while when they are near the C-terminal glycine, the hydrogen atoms from the methyl groups are the ones observed at the closest distances from the geometric center of this residue (Figure 6). These conclusions are supported by the analysis of the distributions of the O–N–GC and O–C–GC angles (Figure 7). TMAO appears to approach the nearest surrounding diglycine molecules less frequently than urea (Figure 6). Depletion of the osmolyte from the nearest vicinity of the peptide does not necessarily have to prejudge the issue of the extent of its influence on the solvation shell of the peptide. This influence may be exerted indirectly, through interactions of the solvation shells in question. The oxygen atom of TMAO is known to have prominent hydrogen-bonding abilities [82]; therefore, it can be expected to influence a number of neighboring water molecules. As discussed above, this atom is most often oriented towards N-terminal glycine in diglycine when TMAO approaches this solute. The experiments suggest that the presence of TMAO in the system has a pronounced enhancing effect on hydrogen bonds in the solvation shell of diglycine. Urea, whose solvation water has different properties than solvation water of diglycine, disturbs it.

The extent of the influence of the presence of the osmolytes on the solvation water of the peptides and the extent of the contact of their solvation shells depend on the composition of the studied systems (Figure 2 and Figures S5–S8). We need to bear that in mind when comparing the results of the simulations with the results of the experiments. This might not be straightforward, because in the case of the simulations, such composition-dependent data are not available. As described in the method section, we studied systems with three osmolyte molecules per one molecule of the peptide to increase the probability of contacts between the osmolyte and the peptide and effectively collect data on preferential orientations and distances of the osmolytes in finite amount of time. Unfortunately, there can also always be discrepancies that arise from imperfections of the models.

Overall, the results of simulations support the view that denaturing osmolytes such as urea accumulate in the vicinity of proteins [34]. They are not conflicting with the models that put forward that urea interacts with both the backbone and side chains [83] and that urea interacts with hydrophobic and hydrophilic groups of proteins [84].

Generally, TMAO is believed to be excluded from the protein backbone [21,34,36]. This promotes burial of the backbone and hence the folding of the polypeptide chain [22]. Cho et al. [85] observed that TMAO interacts with backbone amides of solvent-exposed peptides and they proposed that this process was entropically destabilizing for the unfolded conformations of polypeptide chains—in the folded state, amide groups become buried in the structure, and hence are no longer available to TMAO. We might see some indications that sometimes such interactions might occur between NAGMA and TMAO in the small bulges at the beginning of the radial distribution functions for oxygen atoms of TMAO (Figure 6).

## 4. Conclusions

In this work, we presented the results of investigations on the effect of stabilizing osmolyte–TMAO and destabilizing–urea on the hydration spheres of two peptides: NAGMA and diglycine. A recently developed method [53] allowed us to extract this part of water that is simultaneously influenced by peptide and osmolyte. We characterized the structural and energetic organization of water around the peptides to determine how it changes in the presence of the urea or TMAO.

The introduction of TMAO to the peptide solutions strengthens the water structure around both peptides. However, the effect observed in relation to diglycine is much stronger. Moreover, the mechanism of TMAO interaction is different in both cases. Both TMAO and diglycine generate very strong hydrogen bonds in their surroundings, stronger than in pure water. The interaction of the enhanced hydration spheres of both solutes with similar properties leads to an additional strengthening of hydrogen bonds in the hydration sphere of diglycine. In the case of the NAGMA–TMAO system, the interaction of the hydration spheres of both solutes is very rare. The slight contact of the strongly enhanced TMAO hydration sphere and to a lesser extent enhanced NAGMA hydration sphere causes the strengthening of water hydrogen bonds around NAGMA. During this interaction, as the result of molecular dynamics suggest, the TMAO molecule is oriented with its methyl groups towards the peptide, showing no preferences for specific parts of NAGMA.

In contrast to the stabilizing osmolyte, the effect of urea is significantly different. We have shown that urea disturbs the additional water molecules in diglycine solution and makes them similar to pure water. There is a weak interaction between the spheres of both solutes, and the result of molecular dynamics indicates that it may involve water molecules interacting with the carbonyl group of urea and those interacting with the amino group of diglycine. The situation is completely different in the case of NAGMA, where urea and its hydration sphere is incorporated into the NAGMA hydration sphere. As a result of this interaction, the enhanced NAGMA hydration sphere becomes enriched with two populations of water hydrogen bonds: weakened and strengthened. Due to the slightly higher contribution of the latter, water molecules in the presence of urea form, on average, stronger hydrogen bonds than in the NAGMA hydration shell, and as a result, the water structure around NAGMA is slightly strengthened.

Research on simple models is necessary to study folding phenomena involving larger proteins. Our results showed that the compatibility of the hydration spheres of both solutes (peptide and osmolyte) in terms of energy and structure favors their interactions. Thus, TMAO has a greater effect on diglycine, while urea has a greater effect on NAGMA. Accordingly, both peptides can be used to model different protein fragments: NAGMA as the protein backbone model and diglycine as a model of protein model with polar side chains. Significant differences in the hydration patterns of both peptides seem to be crucial for osmolyte–peptide/protein interactions. Even if direct interactions play a significant role in the case of large proteins, it is the water that facilitates or impedes them.

## Figures and Tables

**Table 1:** The parameters of the HDO band of shared or excess affected water in peptide–osmolyte systems for 1:1 mole fraction, peptide-affected water, osmolyte-affected water, and the bulk water, with corresponding mole fraction of osmolyte, and the respective intermolecular oxygen–oxygen distances. $R_{OO}$ errors have been estimated on the basis of the HDO bands position errors. ^*a*^ Band position at maximum (cm${}^{-1}$). ^*b*^ Band position at the gravity center (cm${}^{-1}$). ^*c*^ Full width at half-height (cm${}^{-1}$). ^*d*^ Integrated intensity (dm${}^{3}\cdot$mol${}^{-1}\cdot$cm${}^{-1}$). ^*e*^ The most likely O⋯O distance (Å). ^*f*^ Mean O⋯O distance (Å).

Solute	ν ^o^_OD_^*a*^	ν ^g^_OD_^*b*^	fwhh ^*c*^	I ^*d*^	R^*o*^_OO_ ^*e*^	R^*g*^_OO_ ^*f*^
pure solutes						
diglycine	2492 ± 2	2454 ± 2	209 ± 4	9100	2.777 ± 0.003	2.803 ± 0.003
NAGMA	2511 ± 2	2485 ± 2	179 ± 4	8914	2.823 ± 0.003	2.833 ± 0.003
urea	2515 ± 2	2496 ± 2	153 ± 4	7708	2.836 ± 0.003	2.844 ± 0.003
TMAO	2486 ± 2	2445 ± 2	211 ± 4	11204	2.793 ± 0.003	2.795 ± 0.003
bulk water	2509 ± 2	2496 ± 2	162 ± 4	10053	2.823 ± 0.003	2.844 ± 0.003
shared or excess affected water						
diglycine–urea	2507 ± 2	2487 ± 2	162 ± 4	2481	2.821 ± 0.003	2.836 ± 0.003
diglycine–TMAO	2416 ± 2	2381 ± 2	153 ± 4	786	2.747 ± 0.003	2.729 ± 0.003
NAGMA–urea	2522 ± 2	2472 ± 2	161 ± 4	778	2.836 ± 0.003	2.818 ± 0.003
NAGMA–TMAO	2438 ± 2	2432 ± 2	193 ± 4	426	2.767 ± 0.003	2.775 ± 0.003

## Data Availability

All the obtained information are available per request.

## Supplementary Materials

Supplementary Materials are available online at https://www.mdpi.com/article/10.3390/ijms22179350/s1.

## Abbreviations

The following abbreviations are used in this manuscript:

AcOEt	ethyl acetate
aNCI	average noncovalent interactions
CDI	1,1${}^{\prime }$-carbonyldiimidazole
DMF	dimethylformamide
DMSO	dimethyl sulfoxide
FTIR	Fourier transform infrared
GC	geometric center
GdmCl	guanidinium chloride
MD	molecular dynamics
MeCN	acetonitrile
NAAMA	*N*-acetyl-alanine-methylamide
NAGMA	*N*-acetyl-glycine-methylamide
NATA	*N*-acetyl-tryptophan-amide
NCI	noncovalent interactions
PTFE	polytetrafluoroethylene
REMD	replica exchange molecular dynamics
TMAO	trimethylamine *N*-oxide
