# Supplementary material for: Hydration of Simple Model Peptides in Aqueous Osmolyte Solutions
Source: Int J Mol Sci. 2021 Aug 28;22(17):9350. doi: 10.3390/ijms22179350 (PMC8431001; doi:10.3390/ijms22179350)
Supplement: Supplementary file 1 [file ijms-22-09350-s001.zip › ijms-1313821-supplementary.pdf]

**Supplementary Material**

# Hydration of simple model peptides in aqueous osmolyte solutions

Aneta Panuszko,[*a] Maciej Pieloszczyk,[a] Anna Kuffel,[a] Karol Jacek,[a] Karol A. Biernacki,[b] Sebastian Demkowicz,[b] Janusz Stangret[a] and Piotr Bruździak[a]

[a]*Department of Physical Chemistry, Chemical Faculty, Gdańsk University of Technology*

*Narutowicza 11/12, 80-233 Gdansk, Poland*

[b]*Department of Organic Chemistry, Chemical Faculty, Gdańsk University of Technology*

*Narutowicza 11/12, 80-233 Gdansk, Poland*

[*] *Corresponding Author E-mail:* aneta.panuszko@pg.edu.pl

## S1. FTIR spectroscopy results

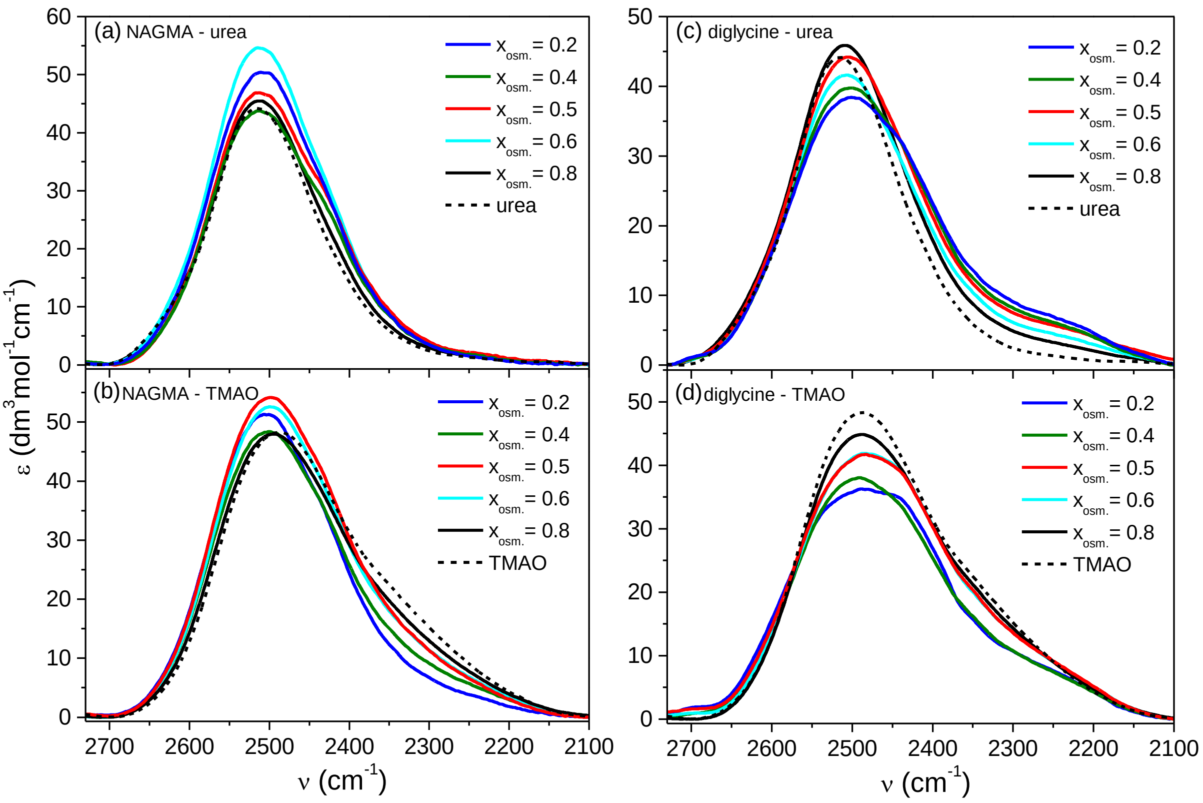


**Figure S1.** Spectra of water affected by peptide and osmolyte in a mixture (experimental affected spectra) for (a, b) NAGMA–osmolyte and (c, d) diglycine–osmolyte systems together with the osmolyte-affected HDO spectrum.

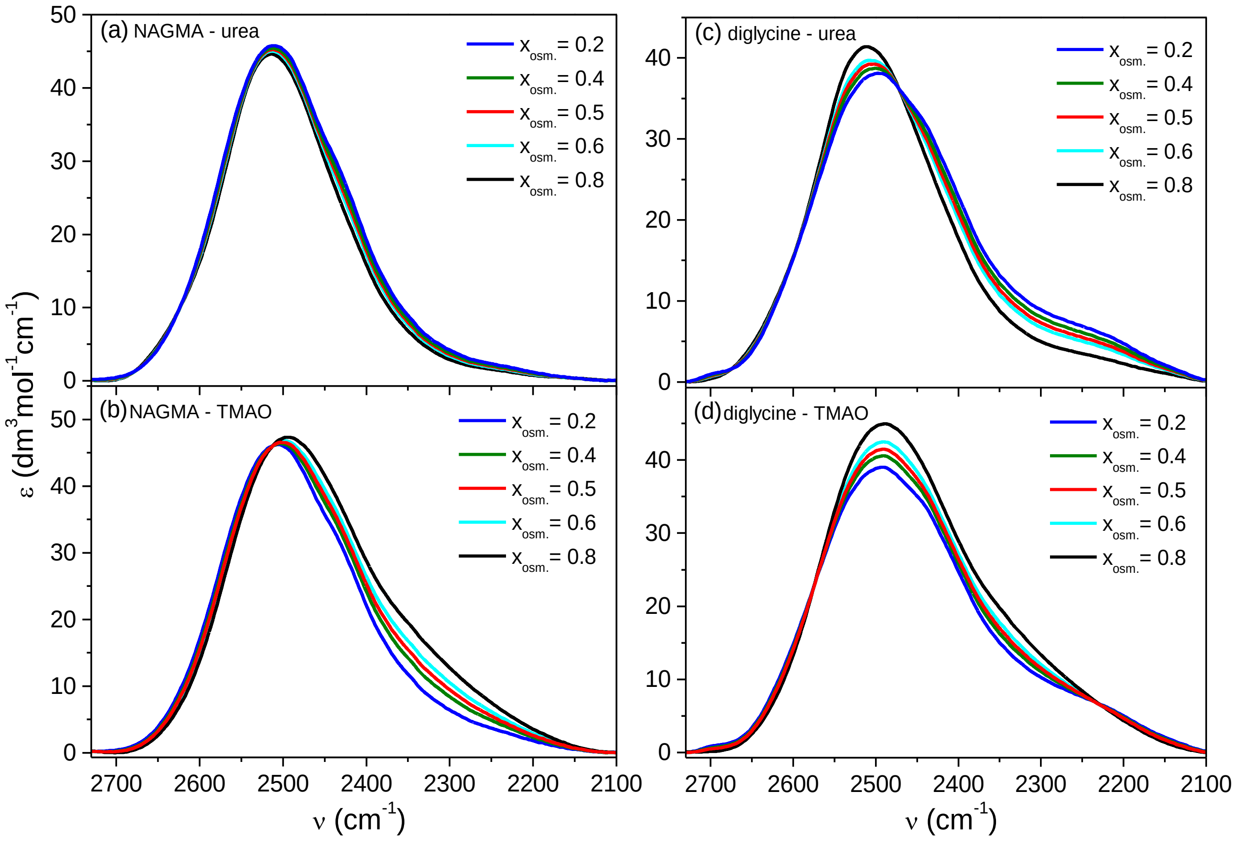


**Figure S2.** Theoretical affected spectra for (a, b) NAGMA–osmolyte and (c, d) diglycine–osmolyte systems.

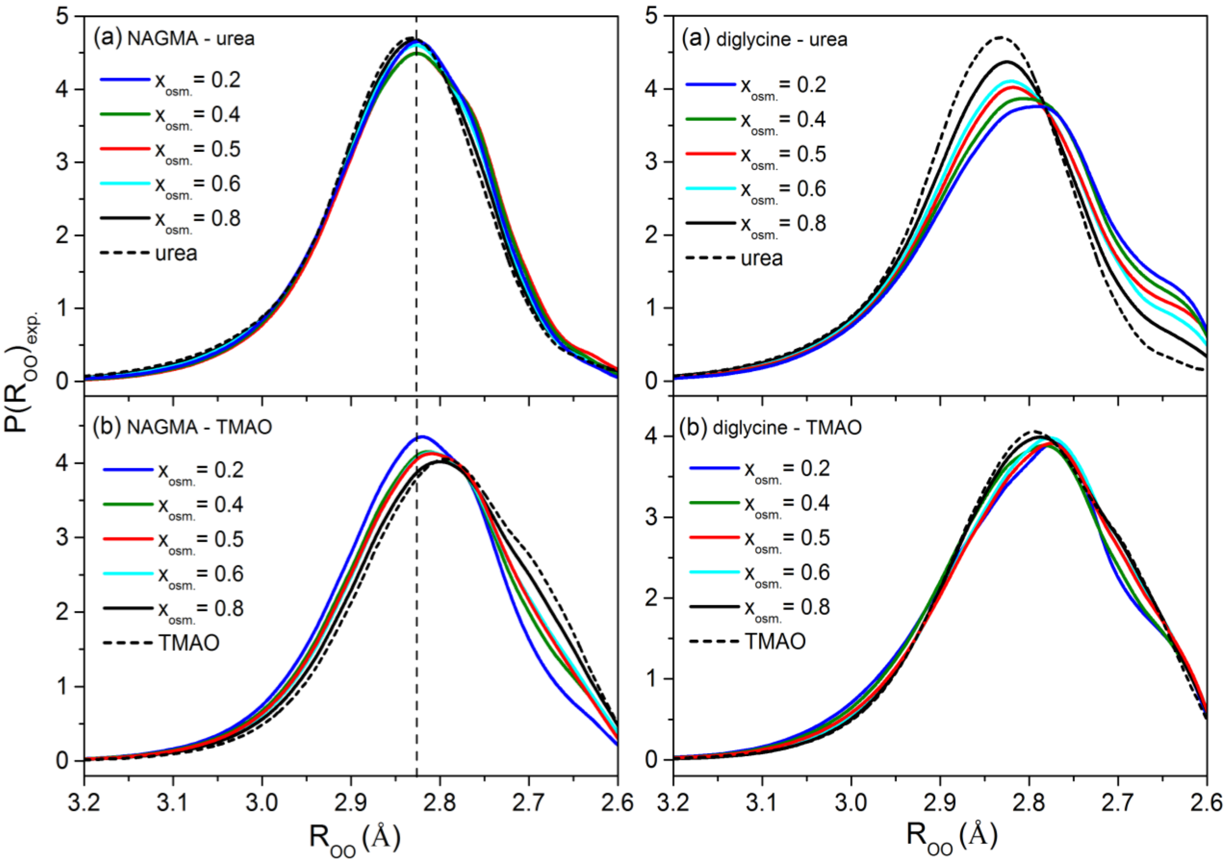


**Figure S3.** Interatomic oxygen–oxygen distance distributions function obtained on the basis of the experimental affected spectra (shown in Fig. S1). The vertical dashed line corresponds to the value of the most likely oxygen–oxygen distance in bulk water (2.823 Å, see Table S1).

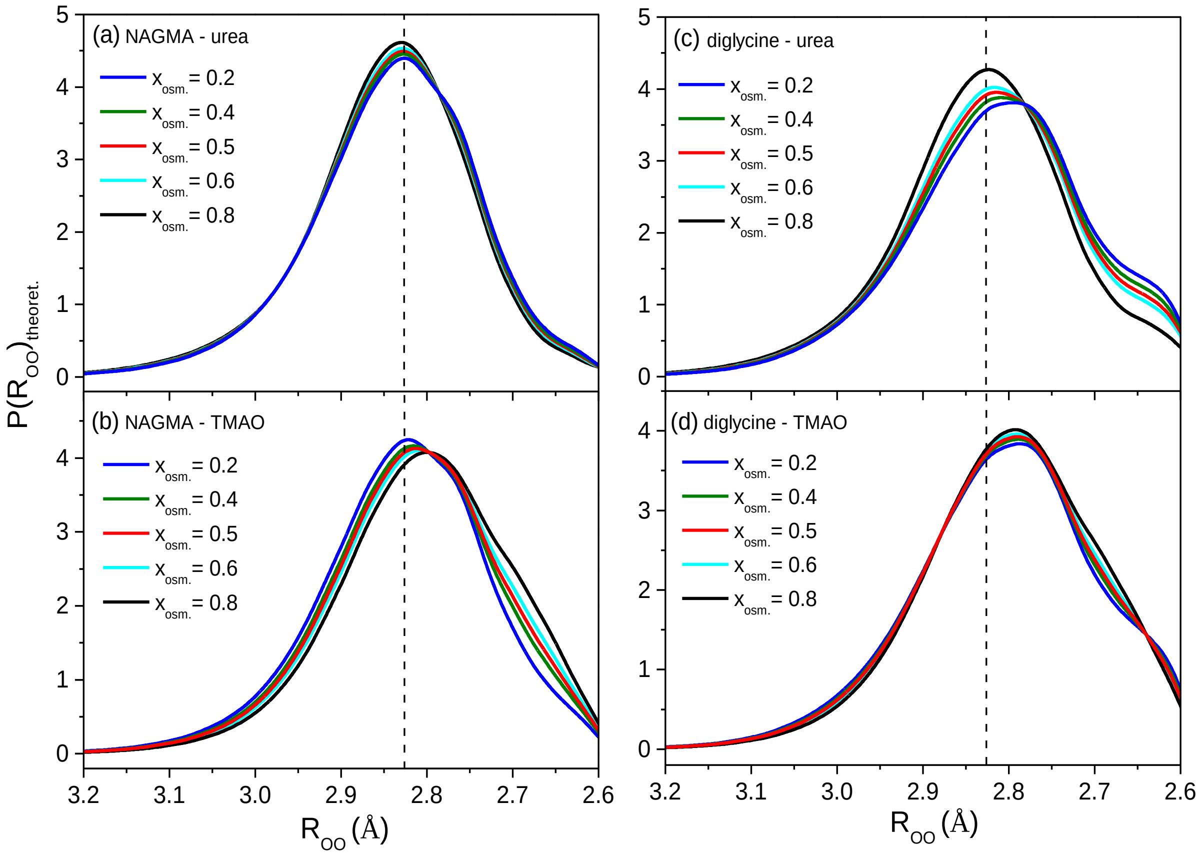


**Figure S4.** Interatomic oxygen–oxygen distance distributions function obtained on the basis of theoretical affected spectra (shown in Fig. S2). The vertical dashed line corresponds to the value of the most likely oxygen–oxygen distance in bulk water (2.823 Å, see Table S1).

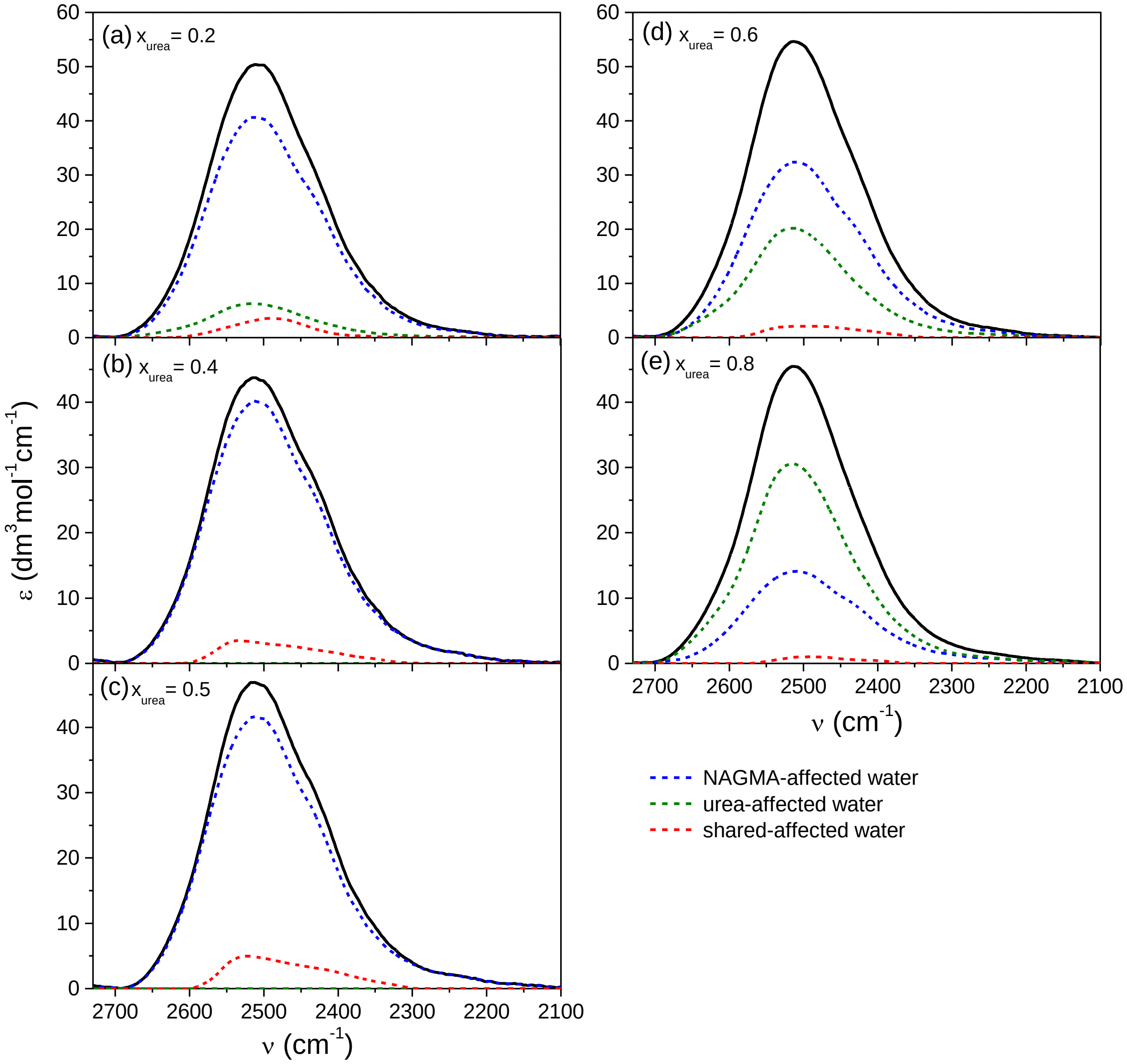


**Figure S5.** The spectra of water affected by NAGMA and urea (experimental affected spectra) (from Fig. S1a) for corresponding mole fraction of urea with isolated contribution of shared affected water, NAGMA-affected water and urea-affected water.

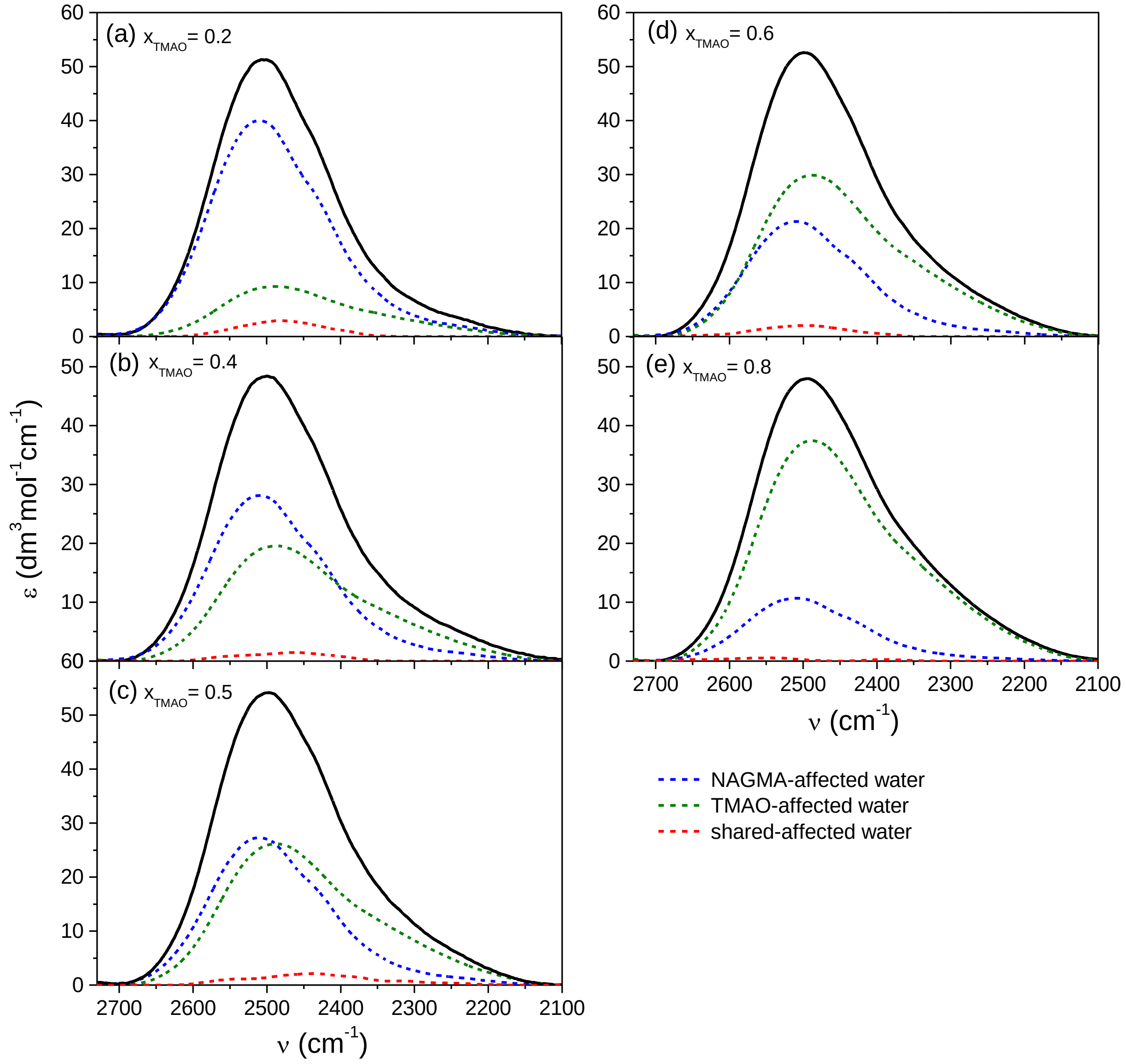


**Figure S6.** The spectra of water affected by NAGMA and TMAO (experimental affected spectra) (from Fig. S1b) for corresponding mole fraction of TMAO with isolated contribution of shared affected water, NAGMA-affected water and TMAO-affected water.

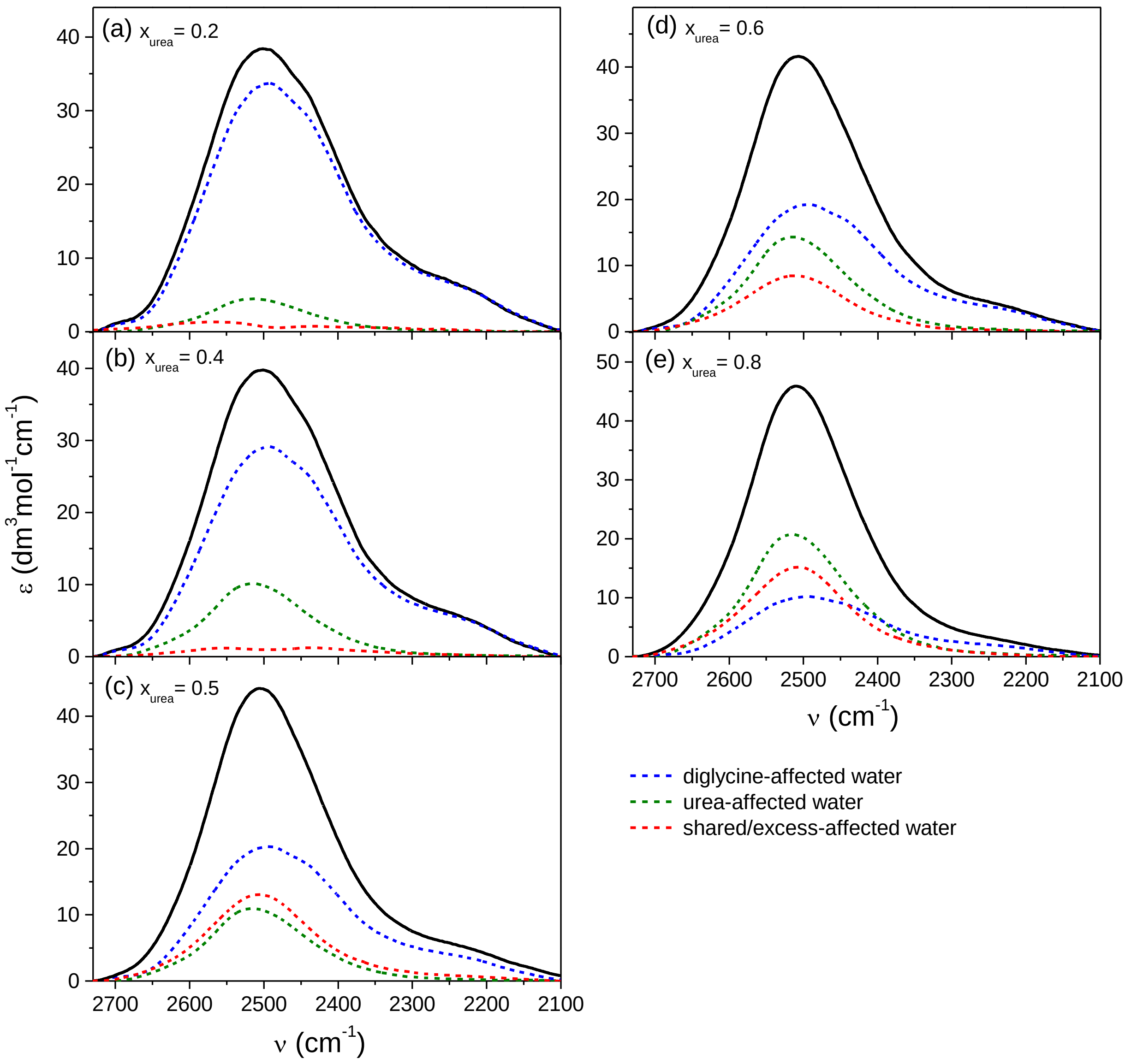


**Figure S7.** The spectra of water affected by diglycine and urea (experimental affected spectra) (from Fig. S1c) for corresponding mole fraction of urea with isolated contribution of shared or excess affected water, diglycine-affected water and urea-affected water.

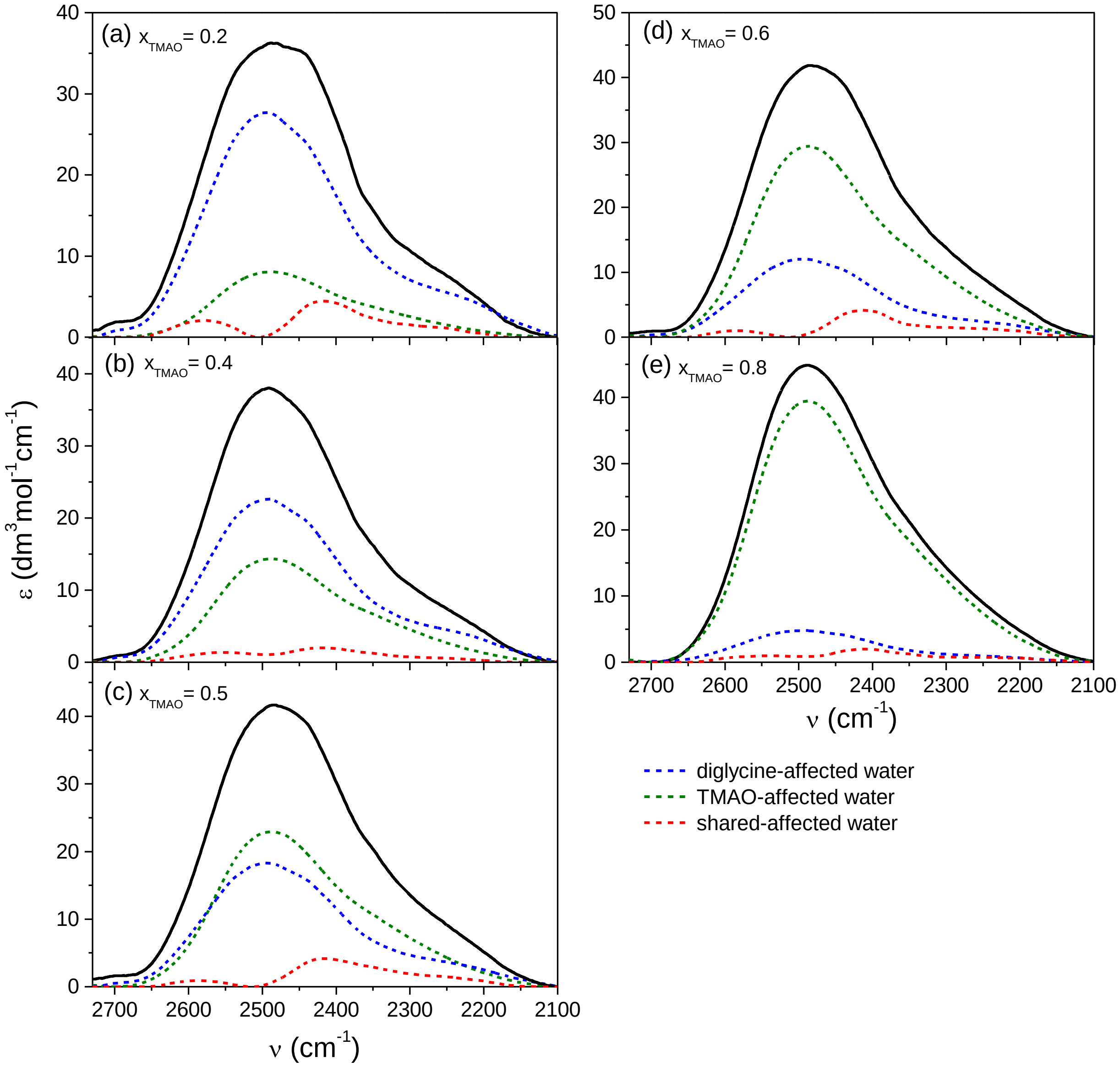


**Figure S8.** The spectra of water affected by diglycine and TMAO (experimental affected spectra) (from Fig. S1d) for corresponding mole fraction of TMAO with isolated contribution of shared affected water, diglycine-affected water and TMAO-affected water.

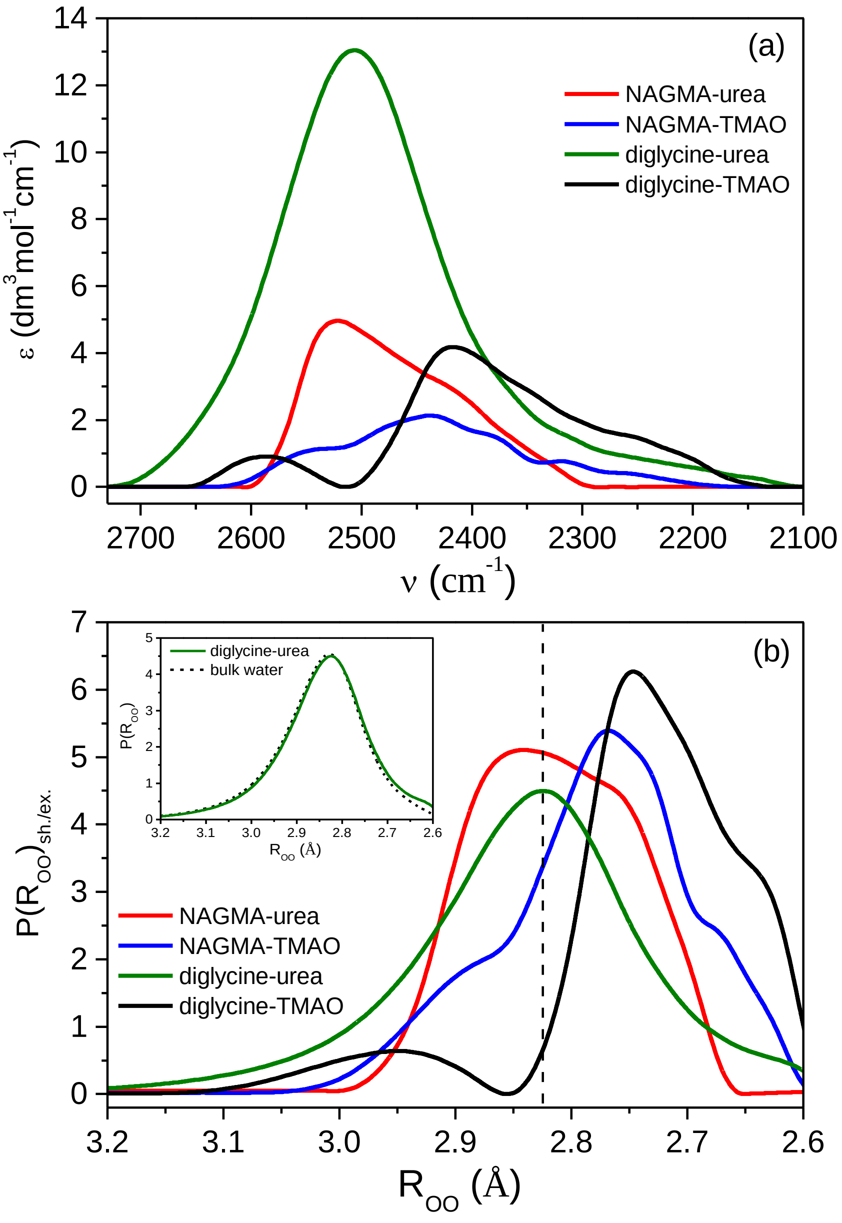


**Figure S9. (a)** The spectra of shared or excess affected water (from Figs. S5c-S8c) in peptide–osmolyte systems for 1:1 mole fraction. **(b)** Interatomic oxygen–oxygen distance distributions function obtained on the basis of the spectra shown in Fig. S9a. The vertical dashed line corresponds to the value of the most likely oxygen–oxygen distance in bulk water (2.823 Å, see Table S1). Inset figure: comparison of the oxygen–oxygen distance distributions obtained for excess water in diglycine–urea system and bulk water.

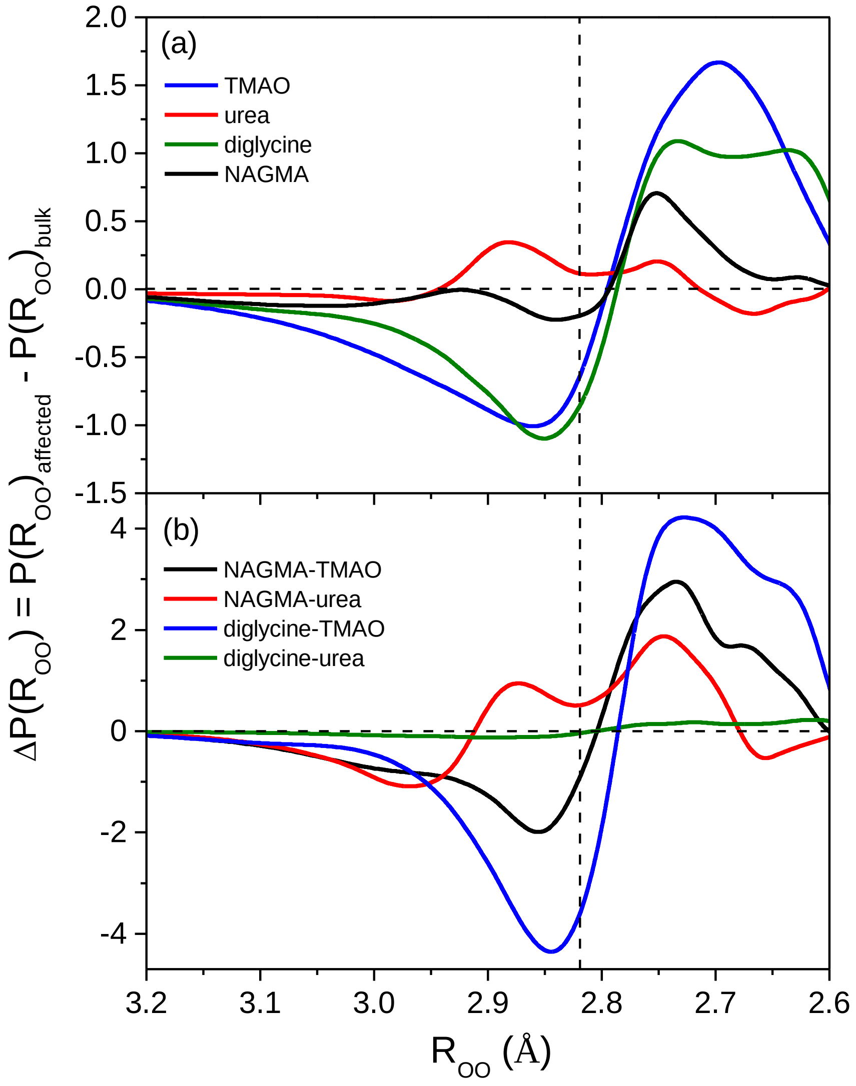


**Figure S10.** Differences between interatomic oxygen–oxygen distance distribution function of: **(a)** water affected by pure solutes: peptides (from Fig. 1b) and osmolytes (from Fig. S3), $P(R_{OO})_{affected}$, and the bulk water (from Fig. 1b), $P(R_{OO})_{bulk}$, **(b)** water affected simultaneously by peptide and osmolyte for 1:1 mole fraction (shared or excess affected water) (from Fig. S9b), $P(R_{OO})_{sh./ex.}$, and the bulk water (from Fig. 1b), $P(R_{OO})_{bulk}$. The vertical dashed line corresponds to the value of the most likely oxygen–oxygen distance in bulk water (2.823 Å, see Table S1).

**Table S1.** The parameters of HDO band of NAGMA-affected water, urea-affected water, the bulk water, experimental and theoretical affected water in NAGMA–urea systems with corresponding mole fraction of osmolyte, and the respective intermolecular oxygen–oxygen distances. $R_{OO}$ errors have been estimated on the basis of the HDO bands position errors.

| Solute | $N^a$ | $\nu^p_{OD}$ $^b$ | $\nu^g_{OD}$ $^c$ | *fwhh* $^d$ | $I$ $^e$ | $R^o_{OO}$ $^f$ | $R^g_{OO}$ $^g$ |
|---|---|---|---|---|---|---|---|
| | | | *pure solutes* | | | | |
| *NAGMA* | 7.5 | 2511±2 | 2485±2 | 179±4 | 8914 | 2.823±0.003 | 2.833±0.003 |
| *urea* | 4.7 | 2515±2 | 2496±2 | 153±4 | 7708 | 2.836±0.003 | 2.844±0.003 |
| *bulk water* | – | 2509±2 | 2496±2 | 162±4 | 10053 | 2.823±0.003 | 2.844±0.003 |
| | | | *experimental affected water* | | | | |
| $x_{osm}$=0.2 | 8.3 | 2507±2 | 2490±2 | 173±4 | 9200 | 2.823±0.003 | 2.836±0.003 |
| $x_{osm}$=0.4 | 7.8 | 2511±2 | 2487±2 | 178±4 | 8213 | 2.826±0.003 | 2.833±0.003 |
| $x_{osm}$=0.5 | 7.9 | 2512±2 | 2484±2 | 177±4 | 8763 | 2.821±0.003 | 2.831±0.003 |
| $x_{osm}$=0.6 | 7.0 | 2513±2 | 2491±2 | 168±4 | 9947 | 2.823±0.003 | 2.836±0.003 |
| $x_{osm}$=0.8 | 6.0 | 2513±2 | 2492±2 | 163±4 | 8129 | 2.826±0.003 | 2.839±0.003 |
| | | | *theoretical affected water* | | | | |
| $x_{osm}$=0.2 | 8.7 | 2511±2 | 2487±2 | 177±4 | 8732 | 2.823±0.003 | 2.833±0.003 |
| $x_{osm}$=0.4 | 10.6 | 2511±2 | 2488±2 | 172±4 | 8535 | 2.826±0.003 | 2.836±0.003 |
| $x_{osm}$=0.5 | 12.2 | 2511±2 | 2489±2 | 170±4 | 8423 | 2.826±0.003 | 2.836±0.003 |
| $x_{osm}$=0.6 | 9.7 | 2513±2 | 2490±2 | 169±4 | 8300 | 2.826±0.003 | 2.839±0.003 |
| $x_{osm}$=0.8 | 6.6 | 2513±2 | 2493±2 | 161±4 | 8016 | 2.826±0.003 | 2.841±0.003 |

$^a$ Affected number, equal to the number of moles of water affected by one mole of solute. $^b$ Band position at maximum ($cm^{-1}$). $^c$ Band position at gravity center ($cm^{-1}$). $^d$ Full width at half-height ($cm^{-1}$). $^e$ Integrated intensity ($dm^3 \cdot mol^{-1} \cdot cm^{-1}$). $^f$ The most likely O···O distance (Å). $^g$ Mean O···O distance (Å).

**Table S2.** The parameters of HDO band of NAGMA-affected water, TMAO-affected water, the bulk water, experimental and theoretical affected water in NAGMA–TMAO systems with corresponding mole fraction of osmolyte, and the respective intermolecular oxygen–oxygen distances. $R_{OO}$ errors have been estimated on the basis of the HDO bands position errors.

| Solute | $N^a$ | $\nu^o_{OD}$ [b] | $\nu^g_{OD}$ [c] | *fwhh* [d] | $I$ [e] | $R^o_{OO}$ [f] | $R^g_{OO}$ [g] |
|---|---|---|---|---|---|---|---|
| | | | *pure solutes* | | | | |
| *NAGMA* | 7.5 | 2511±2 | 2485±2 | 179±4 | 8914 | 2.823±0.003 | 2.833±0.003 |
| *TMAO* | 6.7 | 2486±2 | 2445±2 | 211±4 | 11204 | 2.793±0.003 | 2.795±0.003 |
| *bulk water* | – | 2509±2 | 2496±2 | 162±4 | 10053 | 2.823±0.003 | 2.844±0.003 |
| | | | *experimental affected water* | | | | |
| $x_{osm}$=0.2 | 8.4 | 2507±2 | 2476±2 | 181±4 | 10161 | 2.821±0.003 | 2.823±0.003 |
| $x_{osm}$=0.4 | 11.1 | 2499±2 | 2466±2 | 188±4 | 10242 | 2.810±0.003 | 2.816±0.003 |
| $x_{osm}$=0.5 | 11.2 | 2497±2 | 2463±2 | 191±4 | 11647 | 2.800±0.003 | 2.810±0.003 |
| $x_{osm}$=0.6 | 10.8 | 2499±2 | 2461±2 | 192±4 | 11346 | 2.810±0.003 | 2.810±0.003 |
| $x_{osm}$=0.8 | 8.2 | 2495±2 | 2453±2 | 204±4 | 10836 | 2.800±0.003 | 2.803±0.003 |
| | | | *theoretical affected water* | | | | |
| $x_{osm}$=0.2 | 9.2 | 2505±2 | 2476±2 | 184±4 | 9317 | 2.821±0.003 | 2.826±0.003 |
| $x_{osm}$=0.4 | 12.0 | 2503±2 | 2468±2 | 190±4 | 9724 | 2.821±0.003 | 2.818±0.003 |
| $x_{osm}$=0.5 | 14.2 | 2503±2 | 2464±2 | 191±4 | 9942 | 2.810±0.003 | 2.813±0.003 |
| $x_{osm}$=0.6 | 11.7 | 2497±2 | 2460±2 | 194±4 | 10170 | 2.810±0.003 | 2.810±0.003 |
| $x_{osm}$=0.8 | 8.6 | 2495±2 | 2452±2 | 201±4 | 10670 | 2.800±0.003 | 2.803±0.003 |

[a] Affected number, equal to the number of moles of water affected by one mole of solute. [b] Band position at maximum ($cm^{-1}$). [c] Band position at gravity center ($cm^{-1}$). [d] Full width at half-height ($cm^{-1}$). [e] Integrated intensity ($dm^3 \cdot mol^{-1} \cdot cm^{-1}$). [f] The most likely O···O distance (Å). [g] Mean O···O distance (Å).

**Table S3.** The parameters of HDO band of diglycine-affected water, urea-affected water, the bulk water, experimental and theoretical affected water in diglycine–urea systems with corresponding mole fraction of osmolyte, and the respective intermolecular oxygen–oxygen distances. $R_{OO}$ errors have been estimated on the basis of the HDO bands position errors.

| Solute | $N$ [a] | $\nu^{o}_{OD}$ [b] | $\nu^{g}_{OD}$ [c] | *fwhh* [d] | $I$ [e] | $R^{o}_{OO}$ [f] | $R^{g}_{OO}$ [g] |
|---|---|---|---|---|---|---|---|
| | | | *pure solutes* | | | | |
| *diglycine* | 11.1 | 2492±2 | 2454±2 | 209±4 | 9100 | 2.777±0.003 | 2.803±0.003 |
| *urea* | 4.7 | 2515±2 | 2496±2 | 153±4 | 7708 | 2.836±0.003 | 2.844±0.003 |
| *bulk water* | – | 2509±2 | 2496±2 | 162±4 | 10053 | 2.823±0.003 | 2.844±0.003 |
| | | | *experimental affected water* | | | | |
| $x_{osm}=0.2$ | 11.9 | 2501±2 | 2459±2 | 205±4 | 9189 | 2.785±0.003 | 2.810±0.003 |
| $x_{osm}=0.4$ | 13.5 | 2501±2 | 2463±2 | 199±4 | 9126 | 2.810±0.003 | 2.813±0.003 |
| $x_{osm}=0.5$ | 19.5 | 2507±2 | 2471±2 | 182±4 | 9298 | 2.821±0.003 | 2.821±0.003 |
| $x_{osm}=0.6$ | 14.5 | 2507±2 | 2475±2 | 183±4 | 8684 | 2.821±0.003 | 2.826±0.003 |
| $x_{osm}=0.8$ | 10.0 | 2511±2 | 2485±2 | 168±4 | 8841 | 2.821±0.003 | 2.833±0.003 |
| | | | *theoretical affected water* | | | | |
| $x_{osm}=0.2$ | 12.3 | 2497±2 | 2458±2 | 205±4 | 8960 | 2.800±0.003 | 2.808±0.003 |
| $x_{osm}=0.4$ | 14.2 | 2501±2 | 2462±2 | 199±4 | 8785 | 2.810±0.003 | 2.813±0.003 |
| $x_{osm}=0.5$ | 16.2 | 2501±2 | 2466±2 | 191±4 | 8650 | 2.810±0.003 | 2.816±0.003 |
| $x_{osm}=0.6$ | 12.1 | 2509±2 | 2469±2 | 187±4 | 8539 | 2.821±0.003 | 2.821±0.003 |
| $x_{osm}=0.8$ | 7.4 | 2511±2 | 2479±2 | 172±4 | 8189 | 2.821±0.003 | 2.828±0.003 |

[a] Affected number, equal to the number of moles of water affected by one mole of solute. [b] Band position at maximum ($cm^{-1}$). [c] Band position at gravity center ($cm^{-1}$). [d] Full width at half-height ($cm^{-1}$). [e] Integrated intensity ($dm^3 \cdot mol^{-1} \cdot cm^{-1}$). [f] The most likely O···O distance (Å). [g] Mean O···O distance (Å).

**Table S4.** The parameters of HDO band of diglycine-affected water, TMAO-affected water, the bulk water, experimental and theoretical affected water in diglycine–TMAO systems with corresponding mole fraction of osmolyte, and the respective intermolecular oxygen–oxygen distances. $R_{OO}$ errors have been estimated on the basis of the HDO bands position errors.

| Solute | $N^a$ | $\nu^p_{OD}$ [b] | $\nu^g_{OD}$ [c] | *fwhh* [d] | $I$ [e] | $R^o_{OO}$ [f] | $R^g_{OO}$ [g] |
|---|---|---|---|---|---|---|---|
| | | | *pure solutes* | | | | |
| *diglycine* | 11.1 | 2492±2 | 2454±2 | 209±4 | 9100 | 2.777±0.003 | 2.803±0.003 |
| *TMAO* | 6.7 | 2486±2 | 2445±2 | 211±4 | 11204 | 2.793±0.003 | 2.795±0.003 |
| *bulk water* | – | 2509±2 | 2496±2 | 162±4 | 10053 | 2.823±0.003 | 2.844±0.003 |
| | | | *experimental affected water* | | | | |
| $x_{osm}$=0.2 | 10.2 | 2488±2 | 2454±2 | 226±4 | 9143 | 2.772±0.003 | 2.800±0.003 |
| $x_{osm}$=0.4 | 14.8 | 2492±2 | 2451±2 | 215±4 | 9192 | 2.777±0.003 | 2.800±0.003 |
| $x_{osm}$=0.5 | 14.1 | 2482±2 | 2444±2 | 227±4 | 10240 | 2.772±0.003 | 2.793±0.003 |
| $x_{osm}$=0.6 | 11.0 | 2482±2 | 2443±2 | 222±4 | 10268 | 2.777±0.003 | 2.793±0.003 |
| $x_{osm}$=0.8 | 8.2 | 2488±2 | 2443±2 | 216±4 | 10640 | 2.785±0.003 | 2.793±0.003 |
| | | | *theoretical affected water* | | | | |
| $x_{osm}$=0.2 | 12.8 | 2492±2 | 2453±2 | 210±4 | 9356 | 2.777±0.003 | 2.803±0.003 |
| $x_{osm}$=0.4 | 15.5 | 2492±2 | 2451±2 | 207±4 | 9662 | 2.785±0.003 | 2.800±0.003 |
| $x_{osm}$=0.5 | 17.8 | 2492±2 | 2450±2 | 207±4 | 9839 | 2.785±0.003 | 2.800±0.003 |
| $x_{osm}$=0.6 | 14.1 | 2492±2 | 2449±2 | 210±4 | 10035 | 2.793±0.003 | 2.800±0.003 |
| $x_{osm}$=0.8 | 9.5 | 2488±2 | 2447±2 | 210±4 | 10491 | 2.793±0.003 | 2.798±0.003 |

[a] Affected number, equal to the number of moles of water affected by one mole of solute. [b] Band position at maximum ($cm^{-1}$). [c] Band position at gravity center ($cm^{-1}$). [d] Full width at half-height ($cm^{-1}$). [e] Integrated intensity ($dm^3 \cdot mol^{-1} \cdot cm^{-1}$). [f] The most likely O···O distance (Å). [g] Mean O···O distance (Å).

**Table S5.** Experimental and theoretical numbers of affected water molecules (*N*) for peptide–osmolyte system

| $x_{osm.}$ [a] | $n_{osm.}/n_{pept.}$ [b] | $N_{exp.}$ [c] | $N_{osm., theor.}$ [d] | $N_{pept., theor.}$ [d] | $N_{theor.}$ [e] | $\Delta N$ [f] |
|---|---|---|---|---|---|---|
| | | | *NAGMA–urea system* | | | |
| 0.0 | - | 7.5 | - | 7.5 | 7.5 | - |
| 0.2 | 0.25 | 8.3 | 1.2 | 7.5 | 8.7 | -0.4 |
| 0.4 | 0.67 | 7.8 | 3.1 | 7.5 | 10.6 | -2.8 |
| 0.5 | 1.00 | 7.9 | 4.7 | 7.5 | 12.2 | -4.3 |
| 0.6 | 1.50 | 7.0 | 4.7 | 5.0 | 9.7 | -2.7 |
| 0.8 | 4.00 | 6.0 | 4.7 | 1.9 | 6.6 | -0.6 |
| 1.0 | - | 4.7 | 4.7 | - | 4.7 | - |
| | | | *NAGMA–TMAO system* | | | |
| 0.0 | - | 7.5 | - | 7.5 | 7.5 | - |
| 0.2 | 0.25 | 8.4 | 1.7 | 7.5 | 9.2 | -0.8 |
| 0.4 | 0.67 | 11.1 | 4.5 | 7.5 | 12.0 | -0.9 |
| 0.5 | 1.00 | 11.2 | 6.7 | 7.5 | 14.2 | -3.0 |
| 0.6 | 1.50 | 10.8 | 6.7 | 5.0 | 11.7 | -0.9 |
| 0.8 | 4.00 | 8.2 | 6.7 | 1.9 | 8.6 | -0.4 |
| 1.0 | - | 6.7 | 6.7 | - | 6.7 | - |
| | | | *diglycine–urea system* | | | |
| 0.0 | - | 11.1 | - | 11.1 | 11.1 | - |
| 0.2 | 0.26 | 11.9 | 1.2 | 11.1 | 12.3 | -0.4 |
| 0.4 | 0.66 | 13.5 | 3.1 | 11.1 | 14.2 | -0.7 |
| 0.5 | 1.07 | 19.5 | 5.1 | 11.1 | 16.2 | 3.3 |
| 0.6 | 1.51 | 14.5 | 4.7 | 7.4 | 12.1 | 2.4 |
| 0.8 | 4.07 | 10.0 | 4.7 | 2.7 | 7.4 | 2.6 |
| 1.0 | - | 4.7 | 4.7 | - | 4.7 | - |
| | | | *diglycine–TMAO system* | | | |
| 0.0 | - | 11.1 | - | 11.1 | 11.1 | - |
| 0.2 | 0.25 | 11.9 | 1.7 | 11.1 | 12.8 | -2.6 |
| 0.4 | 0.66 | 13.5 | 4.4 | 11.1 | 15.5 | -0.7 |
| 0.5 | 0.99 | 19.5 | 6.7 | 11.1 | 17.8 | -3.7 |
| 0.6 | 1.49 | 14.5 | 6.7 | 7.4 | 14.1 | -3.1 |
| 0.8 | 3.95 | 10.0 | 6.7 | 2.8 | 9.5 | -1.3 |
| 1.0 | - | 6.7 | 6.7 | - | 6.7 | - |

[a] Molar fractions of osmolyte in peptide–osmolyte systems. [b] The number of osmolyte molecules per one peptide molecule. [c] Experimental values of *N* number in peptide–osmolyte systems. The parameter was obtained in relation to the solute (peptide or osmolyte) in excess. [d] Theoretical contributions of osmolyte or peptide in the theoretical *N* number. It can be calculated per one mole of osmolyte or peptide (form the point of view of osmolyte or peptide). [e] The theoretical *N* number if the molecules would not interact with each other in any way. [f] The difference between experimental and theoretical *N* numbers in peptide–osmolyte systems ($N_{exp.} - N_{theor.}$).

## S2. Molecular dynamics

### S2.1. Details of the simulations

#### S2.1.1. The molecules of the solutes (diglycine and NAGMA)

The initial conformation of diglycine was prepared by cutting out the necessary atoms from one protein molecule downloaded from Protein Data Bank (PDB ID: 5I4W [57]). The initial conformation of NAGMA was taken from Chemical Entities of Biological Interest (CHEBI:74101 [58]). Initial structures of the solutes were then processed in LEaP, which is a part of Amber molecular dynamics package [70], to add missing atoms. In diglycine, the N-terminal amino group of the first glycine was positively charged and the C-terminal carboxyl group of the second glycine was negatively charged.

#### S2.1.2. Protocols of the simulations

During all production runs for all simulated systems, the particle-mesh Ewald method was used for electrostatic interactions. The lengths of chemical bonds involving hydrogen atoms were fixed using SHAKE. A 1.2 nm cutoff for nonbonding interactions was used. Constant temperature (298 K) was kept by the weak coupling to an external bath ($\tau_T$ = 1.0 ps); constant pressure (1 bar) was kept by the weak coupling method ($\tau_p$ = 1.0 ps). The time step was equal to 0.002 ps. Details of the simulation protocol varied between the systems. Pre-production steps and system-dependent settings are described below.

*Systems with three molecules of the osmolytes (TMAO or urea) in water*

The systems were minimized for 1000 steps during which all atoms of the osmolytes were restrained (the force constant *k* was equal to 10 kcal·mol$^{-1}$ Å$^{-2}$). Then, the systems were equilibrated, initially under NVT conditions for 5 ps (298 K, all atoms of the osmolytes were restrained with *k* = 5 kcal·mol$^{-1}$ Å$^{-2}$, initial velocities of atoms were assigned from Maxwell distribution at 298 K), then under NPT conditions for 20 ps (298 K, 1 bar, all atoms of the osmolytes were restrained with *k* = 5 kcal·mol$^{-1}$ Å$^{-2}$, initial velocities of atoms were once again assigned from Maxwell distribution at 298 K) and finally under NPT conditions for about 200 ps with all restraints turned off. These systems were only used for preliminary assessment of the used force field parameters, the analyzed trajectories covered time equal to about 16 ns.

*Systems with the diglycine or NAGMA molecule in water*

The systems were minimized for 1000 steps during which all atoms of the osmolytes were restrained (the force constant *k* was equal to 50 kcal·mol$^{-1}$ Å$^{-2}$). Then, the systems were equilibrated, initially under NVT conditions for 60 ps (298 K, all atoms of the osmolytes were restrained with *k* = 10 kcal·mol$^{-1}$ Å$^{-2}$, initial velocities of atoms were assigned from Maxwell distribution at 298 K), then under NPT conditions for 1.2 ns (298 K, 1 bar, all atoms of the osmolytes were restrained with *k* = 1 kcal·mol$^{-1}$ Å$^{-2}$, initial velocities of atoms were once again assigned from Maxwell distribution at 298 K) and finally under NPT conditions for about 5.4 ns with all restraints turned off.

The systems equilibrated in such a way provided starting conformations for temperature-replica exchange molecular dynamics simulations, which were performed to analyze conformations adopted by these solutes. An algorithm to generate a set of temperatures for the parallel simulations, proposed by Patriksson and van der Spoel [59], is available at http://folding.bmc.uu.se/remd. The following 54 temperatures were used: 282.15 K, 285.30 K, 288.47 K, 291.66 K, 294.89 K, 298.15 K, 301.44 K, 304.75 K, 308.09 K, 311.46 K, 314.85 K, 318.28 K, 321.73 K, 325.21 K, 328.72 K, 332.27 K, 335.84 K, 339.43 K, 343.06 K, 346.72 K, 350.41 K, 354.13 K, 357.88 K, 361.67 K, 365.49 K, 369.34 K, 373.22 K, 377.13 K, 381.08 K, 385.06 K, 389.09 K, 393.14 K, 397.22 K, 401.34 K, 405.50 K, 409.69 K, 413.91 K, 418.17 K, 422.47 K, 426.80 K, 431.17 K, 435.58 K, 440.03 K, 444.52 K, 449.04 K, 453.63 K, 458.24 K, 462.88 K, 467.56 K, 472.29 K, 477.05 K, 481.85 K, 486.70 K, 491.58 K; with replica exchange attempt every 0.4 ps. Three independent simulations were performed, starting from three different conformations selected from about 13-ns-long simulations following the above-described equilibration. At first, the systems were equilibrated for 6 ps at the selected temperatures, initial velocities of all atoms in each replica were assigned from Maxwell distribution. To prevent rotations around the peptide bond at high temperatures, chirality restraints on the backbone were used. The summed length of the simulations (all 54 replicas of all three independent runs) was equal to about 2.5 μs for diglycine and about 2.6 μs for NAGMA. Plots illustrating the exchanges of the replicas between the temperatures can be found below.

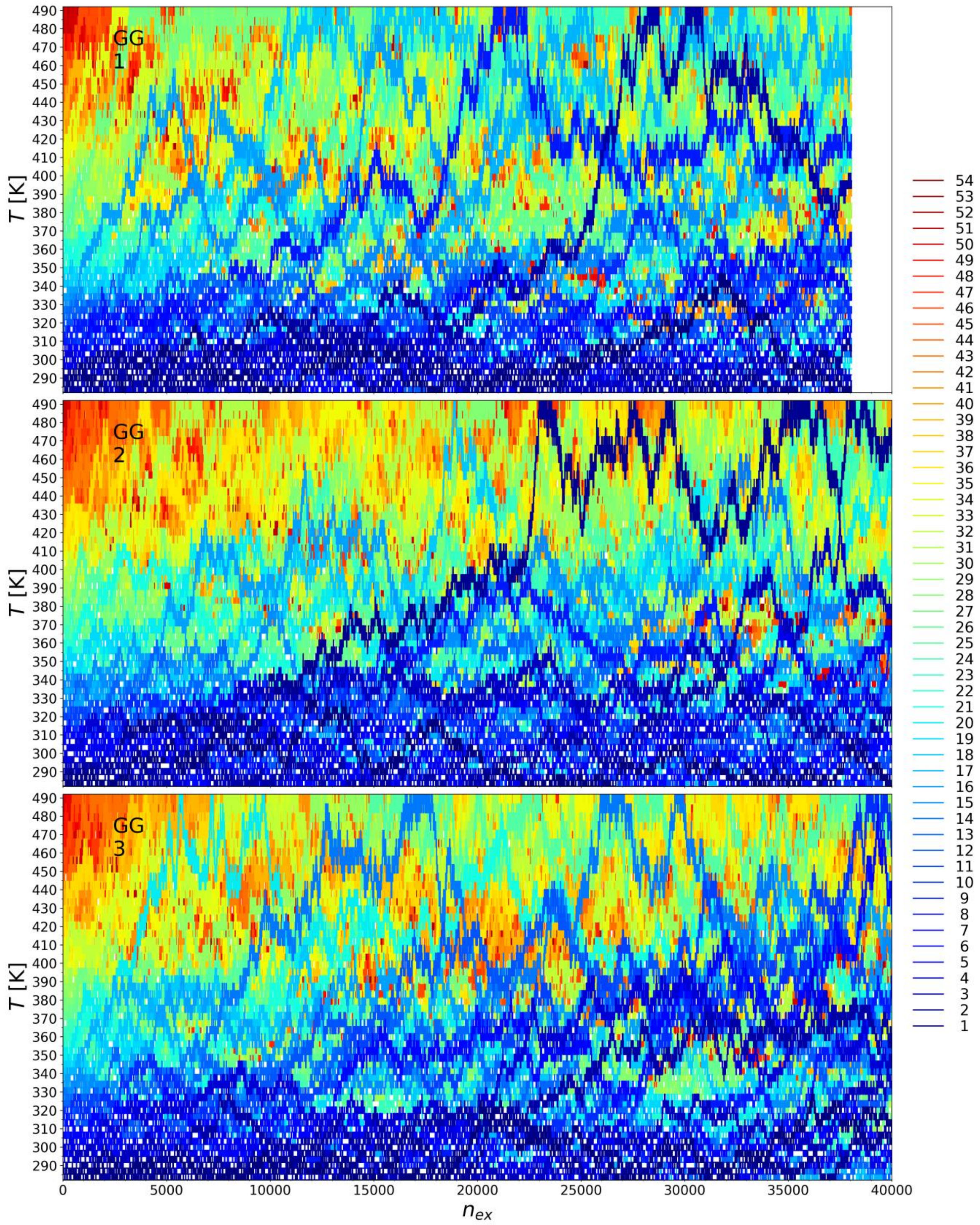


**Figure S11.** The exchange plots of the three independent runs of the temperature-replica exchange molecular dynamics simulations for diglycine in water. 54 coloured lines represent the temperatures ($T$) of the 54 replicas at subsequent exchange attempts ($n_{ex}$).

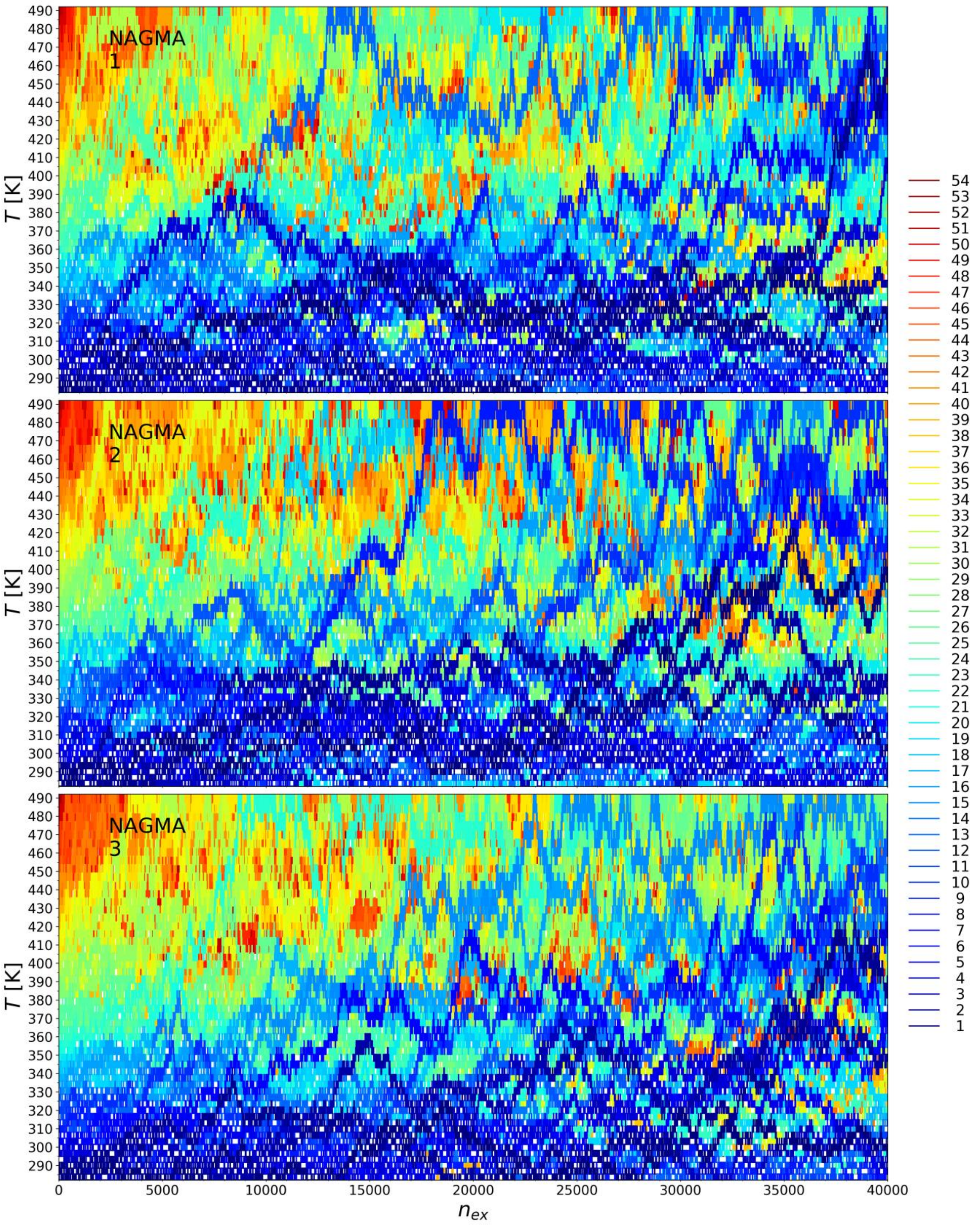


**Figure S12.** The exchange plots of the three independent runs of the temperature-replica exchange molecular dynamics simulations for NAGMA in water. 54 coloured lines represent the temperatures ($T$) of the 54 replicas at subsequent exchange attempts ($n_{ex}$).

The results were analyzed using cpptraj (included in the Amber package), to extract the conformations of the solutes that occurred most frequently at 298.15 K in the obtained trajectories. The most frequently occurring conformation was then used in molecular dynamics simulations to obtain trajectories for weak noncovalent interaction (NCI) analysis, described in section 2.6. They were also used as starting conformations of the solutes in molecular dynamics simulations performed to obtain trajectories for hydrogen bond analysis. The length of the trajectory used during the analysis of properties of hydrogen bonds in peptide solvation shells was equal to about 7.2 ns.

*Systems with the diglycine or NAGMA molecule in water with three molecules of the osmolytes (TMAO or urea)*

These systems were prepared using the conformations of diglycine or NAGMA in water, obtained in the above-described way. Three water molecules were randomly chosen and replaced with molecules of the osmolytes. Ten copies of each system were prepared and simulated independently to improve the efficiency of the analysis. Because the replacement could lead to unfavorable interactions between atoms (resulting in high repulsive force), the systems were carefully inspected during minimization and equilibration. They were minimized for 1000 steps during which all atoms of the osmolytes and heavy atoms of diglycine or NAGMA were restrained (the force constant $k$ was equal to 10 $kcal \cdot mol^{-1}$ $Å^{-2}$). Then, the systems were equilibrated, initially under NVT conditions for 5 ps (298 K, all atoms of the osmolytes and heavy atoms of diglycine or NAGMA were restrained with $k$ = 5 $kcal \cdot mol^{-1}$ $Å^{-2}$, initial velocities of atoms were assigned from Maxwell distribution at 298 K), then under NPT conditions for 20 ps (298 K, 1 bar, all atoms of the osmolytes and heavy atoms of diglycine or NAGMA were restrained with $k$ = 5 $kcal \cdot mol^{-1}$ $Å^{-2}$, initial velocities of atoms were once again assigned from Maxwell distribution at 298 K) and finally under NPT conditions for about 200 ps with all restraints turned off. The summed length of the trajectories used during the analysis of interactions of osmolytes with diglycine or NAGMA was equal to about 1.4 μs.

### S2.2. Results of the analysis of hydrogen bonds

The hydrogen bond between two water molecules was defined using a geometric criterion of Wernet *et al*. [60] with an additional energetic condition - the calculated energy of interaction between two water molecules in question had to be negative. The geometric criterion was defined by the relation $R_{OO} \leq 0.000044 \cdot \beta_{HB}^2 + 0.33$ nm., where $R_{OO}$ is the oxygen−oxygen distance and $\beta_{HB}$ is the O···O−H angle in degrees. This definition was used by us previously [61,62].

Using this definition, we analyzed the changes of number and lengths of hydrogen bonds (defined by the distance O···H between the respective atoms of two hydrogen-bonded water molecules) in the solvation shells of the osmolytes, NAGMA and diglycine – as a function of distance from the analyzed solute, as depicted in figures below (Figures S13, S14 and S15). The figures depict differences of the values of the parameters calculated for solvation water and bulk water filling the same space volume as solvation layer in question. This reference state was obtained by performing additional series of calculations, during which the molecules of the solutes were being inserted into a separately simulated box filled with water molecules, using their momentary coordinates. Next, solvent molecules overlapping with the inserted molecule of the solute were removed. The problem of choosing the right cut-off value for this removal was examined previously [62].

The results suggest that hydrogen bonds between water molecules in solvation shell of TMAO become visibly shorter than in bulk water, while urea influences the distribution of hydrogen bond lengths significantly less (experimental data in Table 1 and Figure S3, molecular dynamics data in Figure S13). The hydrogen bonds also become shorter in solvation layers of both diglycine and NAGMA, but the effect is stronger for diglycine (experimental data in Figure 1, molecular dynamics data in Figure S14).

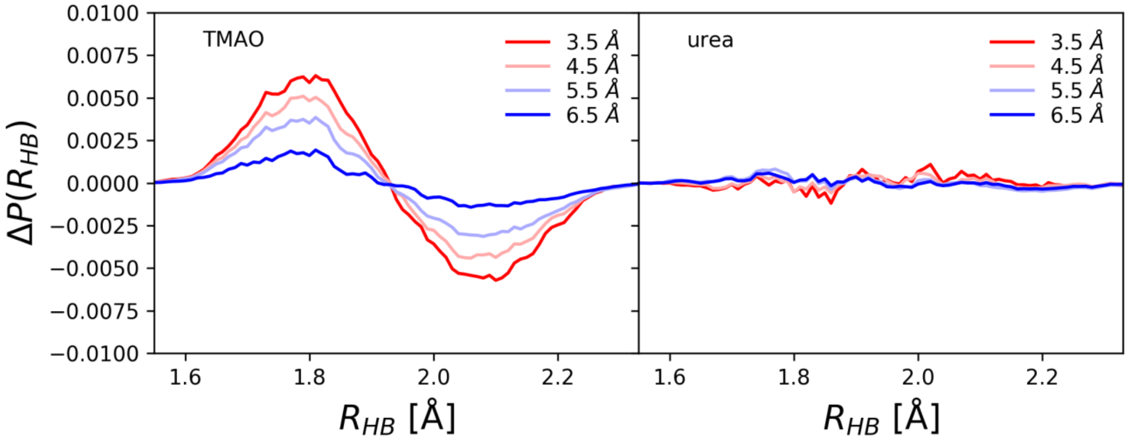


**Figure S13.** The differential histograms (solvation water minus bulk water filling the solvation shells) of lengths of hydrogen bonds ($R_{HB}$) between water molecules in solvation layers of TMAO and urea in systems with three TMAO molecules and three urea molecules in water. The distances given in the legends are the upper limits of the analyzed solvation layers (distances from the solute).

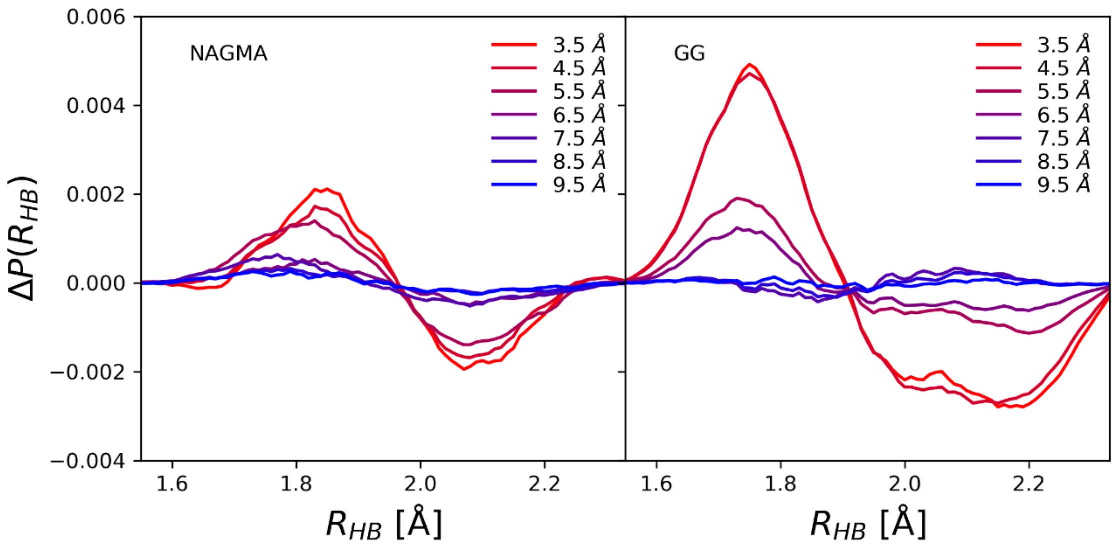


**Figure S14.** The differential histograms (solvation water minus bulk water filling the solvation shells) of lengths of hydrogen bonds ($R_{HB}$) between water molecules in solvation layers of NAGMA and diglycine in systems with one NAGMA molecule and one diglycine molecule in water. The distances given in the legends are the upper limits of the analyzed solvation layers (distances from the solute).

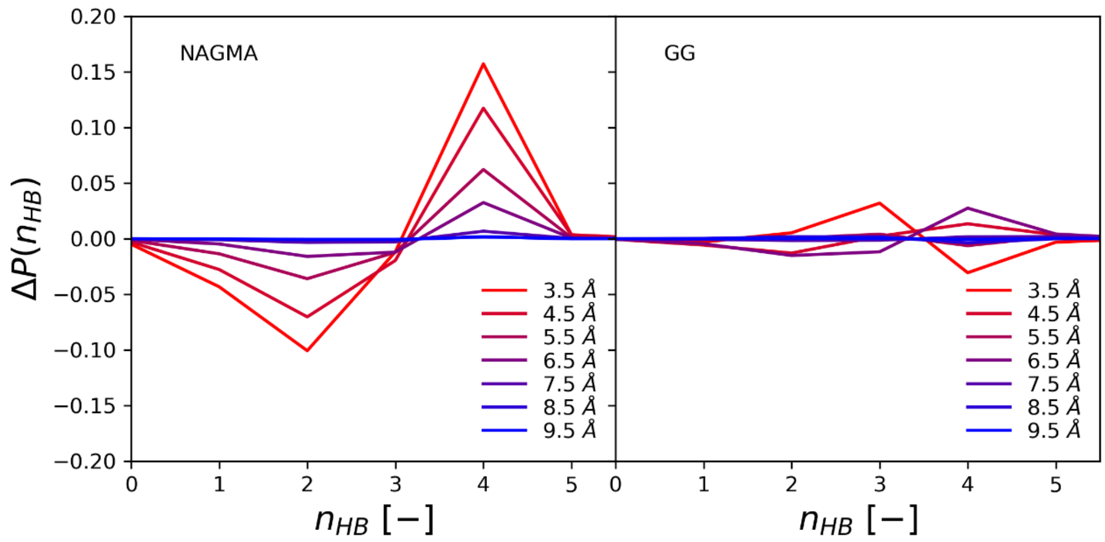


**Figure S15.** The differential histograms (solvation water minus bulk water filling the solvation shells) of number of hydrogen bonds ($n_{HB}$) created by a single water molecule with other water molecules in solvation layers of NAGMA and diglycine in systems with one NAGMA molecule and one diglycine molecule in water. The distances given in the legends are the upper limits of the analyzed solvation layers (distances from the solute).

## S3. NAGMA synthesis

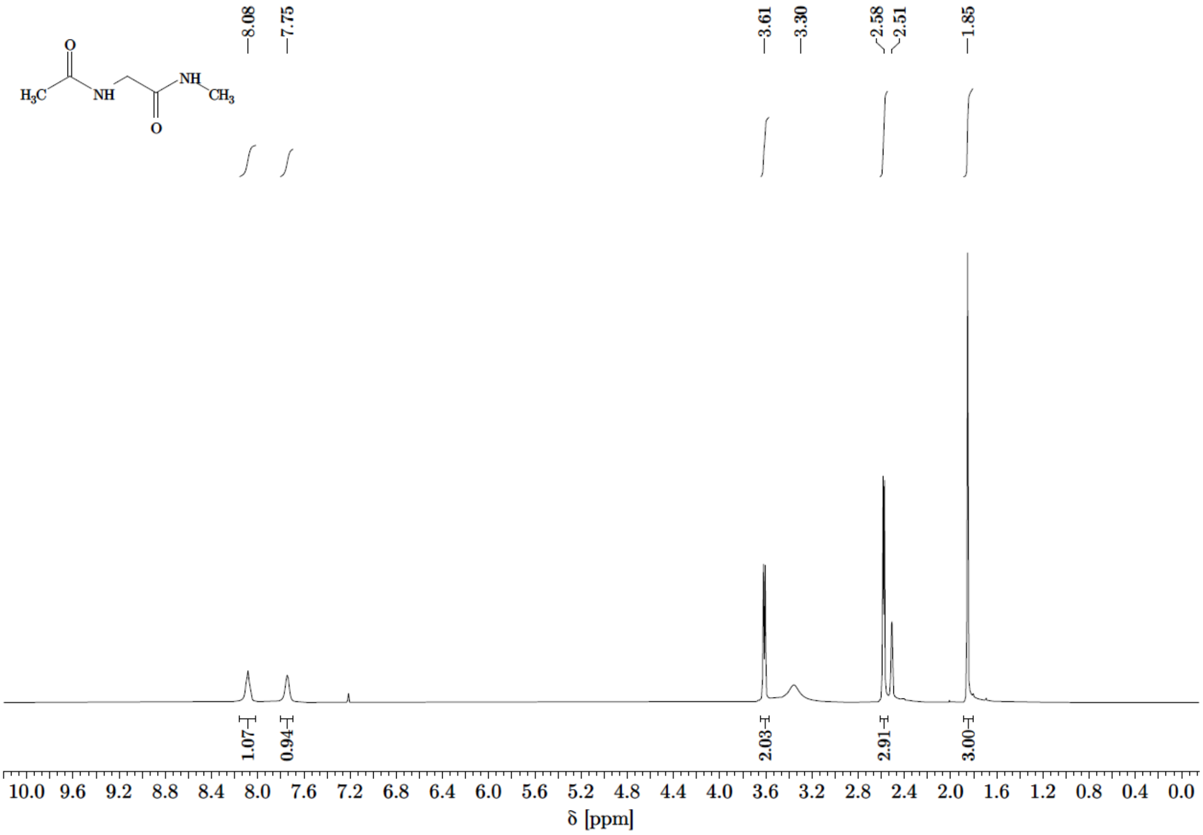


**Figure S16.** $^{1}$H NMR spectrum of NAGMA

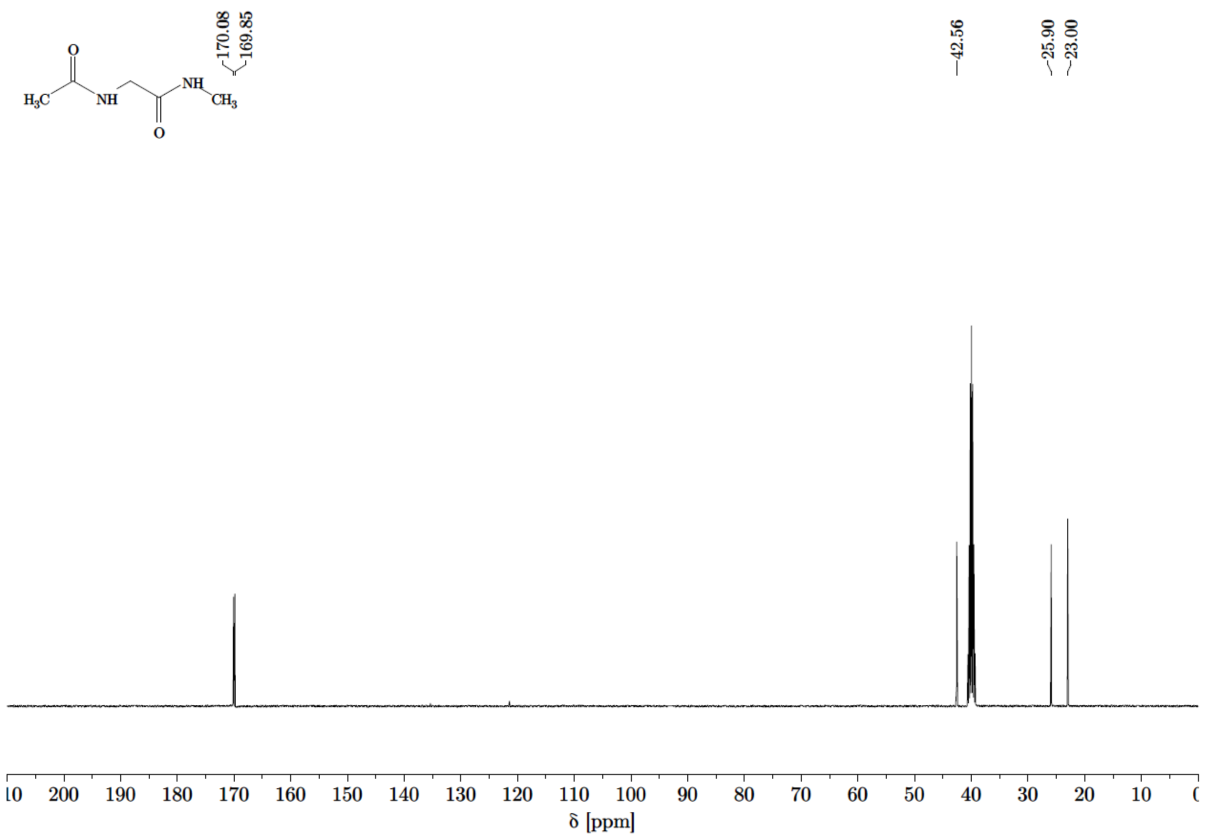


**Figure S17.** $^{13}$C NMR spectrum of NAGMA

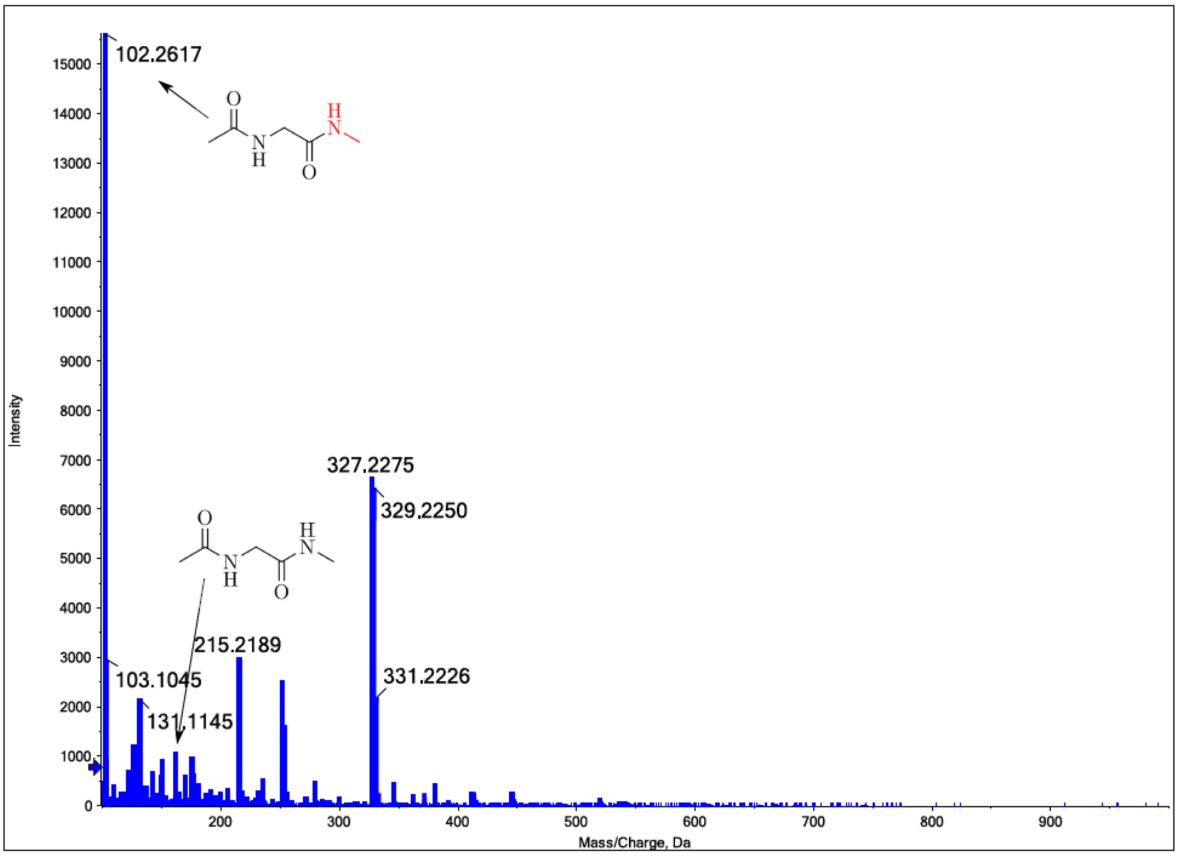


**Figure S18.** The MS spectrum in positive ionization mode of NAGMA and ion identities. The red color indicates the molecular fragments released during fragmentation. The signals observed in the MS spectrum correspond to the molecular fragments shown in black.
